# Tamper-Evident Provenance for Secure IoT Edge Actuation with Signature-Independent On-Chain Commitments

**DOI:** 10.3390/s26196262

**Published:** 2026-10-02

**Authors:** Kuo-Yu Tsai, Yu-Yun Hsieh, Che-Cheng Chang

**Affiliations:** Department of Information Engineering and Computer Science, Feng Chia University, Taichung 407802, Taiwan; p1535652@o365.fcu.edu.tw (Y.-Y.H.); checchang@fcu.edu.tw (C.-C.C.)

**Keywords:** Internet of Things (IoT), IoT security, edge computing, edge actuation, tamper-evident provenance, blockchain, post-quantum cryptography, industrial automation

## Abstract

IoT edge actuation requires records that remain auditable after an incident. We present a crypto-agile provenance framework that keeps decision records and signatures off-chain while a Sui Move contract stores a 32-byte SHA-256 anchor, bounded metadata, and a one-byte scheme identifier. Public keys and their ledger-time validity windows are registered on-chain for historical auditing. For a fixed multi-key endpoint, measured commitment gas is identical across Ed25519, ML-DSA-44, and SLH-DSA-SHA2-128f despite a 267-fold signature-size range; it also remains unchanged across a 3301-fold record-size range. Different endpoint key sets introduce up to 5.66% variation through state-rewrite costs on the evaluated localnet. On a Raspberry Pi 5, the mean of three ML-DSA-44 run medians is 0.65 ms, with a signing-latency coefficient of variation of approximately 78% and substantial upper tails. Additional crossed key-by-message measurements show no nominally significant ML-DSA key-factor result on the Pi, but two nominally significant ML-DSA-44 key-factor results in the desktop sessions, one of which remains significant after Bonferroni correction across 72 comparisons. These results do not establish key independence or a variance upper bound; they support budgeting for observed signing tails while preserving uncertainty about persistent key effects. The framework establishes tamper evidence and conditional endpoint attribution, not physical truthfulness of telemetry or the time a signature was created.

## 1. Introduction

Wireless sensor networks (WSNs) and Internet of Things (IoT) systems increasingly connect sensing, edge processing, and physical actuation within the same cyber–physical workflow. Sensor and edge nodes no longer merely report environmental or operational measurements; they may also use local or remotely supplied information to unlock doors, operate motors, dispense materials, move robotic systems, or execute manufacturing instructions. This transition from monitoring to actuation expands the security objective of sensor-networked systems. In addition to confidentiality, authentication, and reliable data acquisition, post-event analysis may require independently verifiable evidence of which endpoint accepted an actuation task, which digital inputs and reported measurements were associated with the decision, and whether the corresponding record was modified after the event. Authentication, privacy, trust management, and constrained-device security remain recurring IoT concerns [1,2], but actuation adds an accountability requirement that ordinary telemetry alone does not satisfy.

Manufacturing provides a concrete example. A controller can receive a protected design file, satisfy an authorization policy, start fabrication, and report process telemetry while the job runs. If the resulting component later fails inspection, an auditor may need to determine whether the intended design file was used, whether the endpoint reported an anomaly, and whether the available record existed before the dispute. Conventional logs are useful for diagnosis, but a centrally administered database can be modified by an operator with sufficient privileges. A standalone file hash, meanwhile, proves only that a set of bytes matches a digest; it does not establish which registered endpoint asserted those bytes. A useful provenance mechanism therefore needs both source authentication and an external, tamper-evident reference to the signed record.

Blockchain anchoring offers one way to provide such a reference [3,4,5,6,7]. However, a blockchain is a poor place to retain verbose telemetry, long-lived audit bundles, or large digital signatures. Directly storing complete records makes recurring ledger cost grow with record volume. Directly storing signatures also couples the persistent on-chain format to the selected cryptographic scheme. This coupling becomes particularly relevant during post-quantum migration. NIST standardized ML-DSA and SLH-DSA in 2024 [8,9], while Ed25519 remains a widely deployed classical baseline [10]. Post-quantum signatures can be much larger than Ed25519 signatures, so embedding their complete encodings in recurring blockchain state increases storage cost and can encounter ledger-specific transaction-size limits.

This paper presents Edge Attestation Lab, a crypto-agile, post-quantum-ready provenance framework for secure IoT edge actuation. Each endpoint deterministically serializes a decision record and signs the resulting bytes with an attestation key. The full record and signature are retained off-chain, while the evidence bundle carries a copy of the public key. The endpoint’s registered public keys and their ledger-time validity windows are maintained on-chain for historical audit. A Sui Move contract stores a fixed 32-byte SHA-256 commitment together with bounded metadata and a one-byte signature-scheme identifier. Signature verification remains an auditor-side operation. Consequently, a change in endpoint signature family does not introduce a scheme-dependent variable-length signature field into the recurring commitment object.

The work builds on, but is distinct from, the manufacturing authorization framework in [11]. That system determines whether a manufacturing endpoint may obtain the material required to execute a task and enforces the decision cryptographically. Once authorization succeeds, however, it does not cryptographically bind a subsequent endpoint decision record—for example, a design-file hash, telemetry summary, and decision label—to evidence available during a later audit. Execution authorization from [11] is therefore treated here as an external prerequisite and is neither re-implemented nor re-evaluated. The present contribution addresses the post-authorization provenance layer: what did the endpoint record, what did it sign, and when was that signed record anchored?

This distinction also defines the security boundary. The proposed framework preserves and attributes endpoint statements; it does not prove that firmware-reported telemetry represents physical ground truth. A compromised endpoint can sign a false temperature or status value, and the framework will preserve that false claim faithfully. Stronger physical assurance requires complementary mechanisms, such as secure boot, hardware-backed attestation, trusted sensing, or independent observers. Likewise, the application-layer post-quantum signatures evaluated here do not make the underlying blockchain transaction-authentication layer post-quantum secure.

The evaluation addresses three research questions. **RQ1** asks whether recurring on-chain commitment cost can remain independent of signature length and underlying record size when the ledger stores only a fixed-width digest. **RQ2** asks what computation and storage tradeoffs Ed25519, ML-DSA, and SLH-DSA impose on a Raspberry Pi 5-class edge endpoint. **RQ3** asks whether the heavy signing-latency tail observed for ML-DSA contains a persistent key-attributable component that could be mitigated by selecting different keys, and what the measured latency distribution implies for scheduling.

The main contributions are as follows:

- A crypto-agile provenance architecture for IoT sensing-to-actuation workflows that separates endpoint-signed evidence from a fixed-size on-chain commitment, thereby avoiding scheme-dependent signature fields in the recurring commitment object.
- A Rust and Sui Move implementation supporting deterministic provenance records, governed signature-scheme registration, per-endpoint key sets, lifecycle controls, scheme migration, and independent off-chain verification.
- Edge-class measurements of Ed25519, ML-DSA, and SLH-DSA signing, verification, public-key size, signature size, and evidence-bundle size on a Raspberry Pi 5, including percentile-based characterization of the ML-DSA signing-latency tail.
- Sui localnet measurements showing identical commitment gas components for the same multi-key endpoint when only the scheme identifier changes, despite a 267-fold spread in off-chain signature size and a 3301-fold range in record size, together with a separate experiment showing at most 5.66% residual variation across endpoints with different registered public-key lengths.
- A crossed key-by-message experiment on two platforms, with an Ed25519 control, that reports all 18 replicates, including two desktop ML-DSA-44 excursions, and distinguishes observed variance estimates from unsupported independence or upper-bound claims.

## 2. Related Work

This work intersects blockchain-based security for IoT and sensor networks, post-quantum cryptography for constrained and edge devices, and cyber–physical manufacturing auditability.

### 2.1. Blockchain-Based Security and Provenance for IoT and Sensor Networks

Blockchain and smart-contract mechanisms have been widely studied as coordination and audit layers for IoT systems [4,5,6,7]. Their attraction in a provenance setting is not that a ledger should hold all sensor data, but that it can provide an externally ordered and append-only reference to selected evidence. Architectural work on blockchain applications similarly emphasizes the separation between on-chain logic and off-chain components [12]. This separation is especially relevant to WSN and IoT deployments, where sensing data can be high volume, network links may be constrained, and secure data management must coexist with practical storage and computation limits.

Recent work illustrates several points in the design space. When post-quantum verification is placed on-chain, transaction cost becomes scheme-dependent: an EVM evaluation reported large gas differences among W-OTS+, XMSS, SPHINCS+, and MAYO [13]. Santos et al. [14] instead expose cryptographic agility at the protocol layer through a signature-type identifier and per-scheme verification pricing. A hash-based commit–reveal alternative removes signatures from the authorization path and derives post-quantum security from hash assumptions, at the cost of a two-phase transaction flow [15]. In Sensors, Sezer [16] presented a post-quantum sensor-to-blockchain framework that uses hash-based commitments and ML-DSA authentication for compact audit recording.

The present work shares the principle of retaining compact commitments rather than raw sensing or actuation records on-chain, but asks a different question: how can the endpoint signature family remain revisable without changing the recurring commitment representation? The system therefore uses a governed one-byte scheme identifier, per-endpoint key sets, and off-chain verification, and experimentally compares commitment cost across multiple signature families. This makes cryptographic agility an explicit application-layer property rather than fixing one post-quantum scheme in the data model.

### 2.2. Post-Quantum Cryptography for Edge and Constrained Devices

NIST FIPS 204 and FIPS 205 standardize ML-DSA and SLH-DSA, respectively [8,9], while the earlier Dilithium and SPHINCS literature provides their algorithmic context [17,18]. IoT-focused surveys have long emphasized that post-quantum migration must be evaluated under device-level computation, memory, and communication constraints [19]. These constraints are particularly important for edge nodes that both process sensor data and make time-sensitive actuation decisions.

The dispersion of ML-DSA signing latency is also relevant to real-time or near-real-time edge operation. The Dilithium specification models retry count as a geometric random variable and argues that its expected distribution is independent of the secret key [20]. Current IETF guidance for constrained devices treats rejection-sampling behavior as a deployment consideration and recommends disclosing the signing mode and measurement methodology [21]. Recent Cortex-M0+ measurements report substantial coefficients of variation for ML-DSA signing [22]. The present work complements those studies with Raspberry Pi 5 measurements and, more specifically, a crossed key-by-message experiment designed to test whether a persistent key-attributable component can be resolved in a deterministic implementation.

### 2.3. Cyber–Physical Manufacturing and Prior Authorization Layer

Industry 4.0 research motivates tight coupling between digital records and physical manufacturing processes [23]. In such settings, a provenance record can become evidence in warranty analysis, safety review, or quality disputes. The closest prior system along the lines of the present research is [11], which implements a smart-contract-based manufacturing authorization framework. Its Move contract verifies endpoint ownership, capability, task existence, and endpoint consistency before a key server releases the decryption key for a protected design. The workflow was validated end to end on a Raspberry Pi 5 connected to a desktop 3D printer.

That work answers the question of whether an endpoint is permitted to act. It does not solve the post-authorization evidence problem considered here. Once key release has occurred, the authorization protocol cannot establish what the endpoint subsequently recorded about the decision or whether a later off-chain record was modified. The present system therefore adds endpoint-signed decision provenance rather than replacing the prior authorization layer. It also leaves the identity-based encryption and the blockchain’s own transaction authentication unchanged.

### 2.4. Positioning of This Work

The architectural distinction is summarized in Table 1. To the best of our knowledge, the combination evaluated here—off-chain endpoint signatures, a fixed-width on-chain commitment, a governed signature-scheme registry, and cross-scheme measurement of recurring commitment cost—has not been evaluated in the same form in the cited systems. The novelty claim is intentionally narrow: the contribution is not a new signature algorithm or a new blockchain consensus protocol, but a crypto-agile provenance pattern and an empirical evaluation of where cryptographic size and latency costs do, and do not, appear in an IoT edge actuation workflow.

## 3. System Model and Threat Model

### 3.1. Architecture and Trust Boundaries

**Figure 1:**
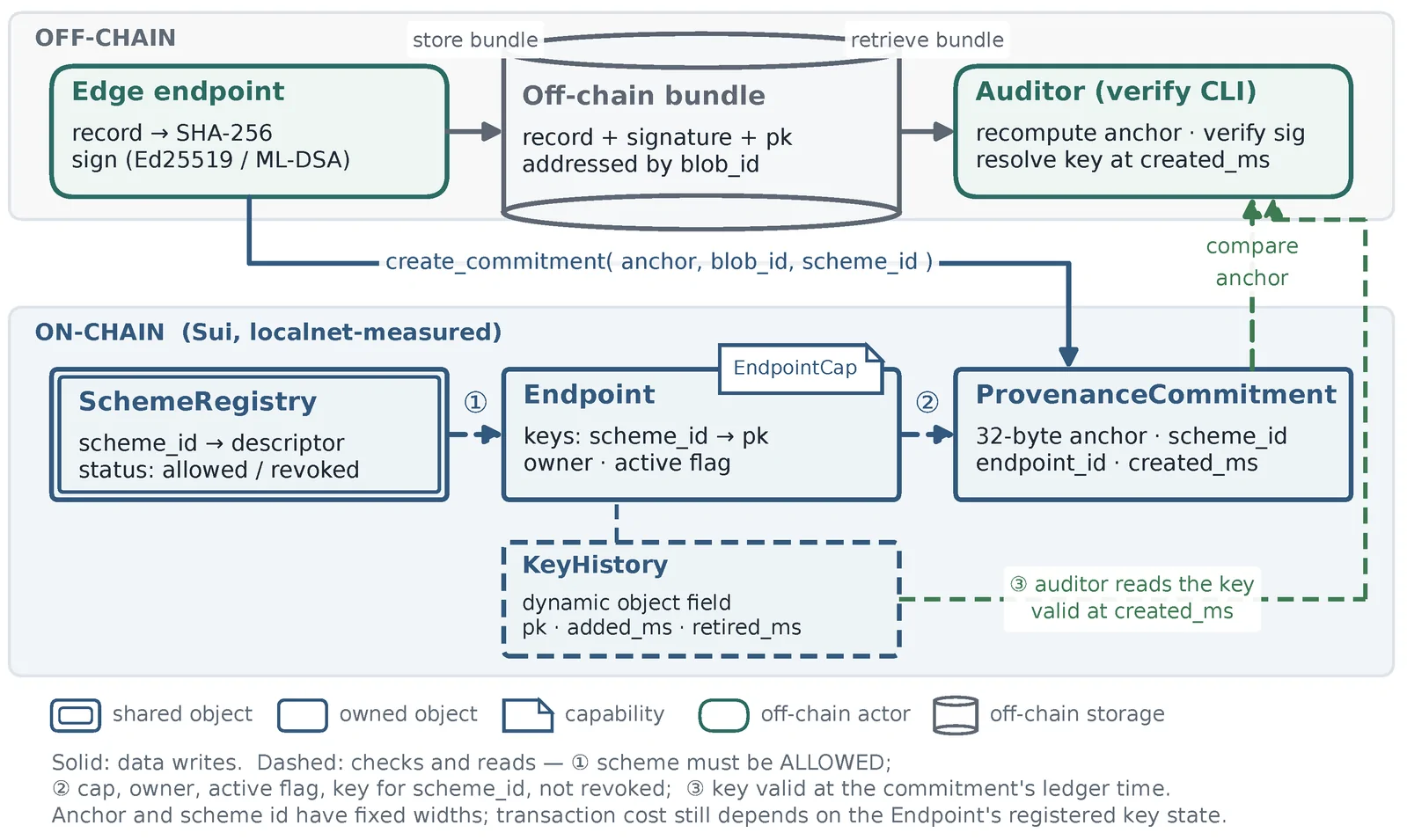
Edge attestation data path after the scheme-registry refactor. Records and signatures remain off-chain in a bundle addressed by blob id. The chain stores scheme state, endpoint public keys and their validity windows, and a 32-byte anchor per decision; the public keys are registered per endpoint and scheme and do not enter the commitment object, while their length can still affect recurring transaction cost through the endpoint-object rewrite. The auditor resolves the key the endpoint held at the commitment’s ledger timestamp rather than the key it holds now, reading the retained KeyHistory attached to the endpoint as a dynamic object field. Dashed edges mark checks and reads: the two numbered checks are enforced at registration and commitment time, the key-history read at audit time.

The method separates a physical actuation record into off-chain evidence and an on-chain commitment. The architecture contains four actors: an edge endpoint, an off-chain record store, a Move contract, and an auditor. The endpoint observes or receives an actuation task, samples telemetry, and constructs a provenance record. The record is serialized into deterministic bytes so that any auditor can reproduce the same hash. The endpoint signs those bytes, stores the signed bundle off-chain, and sends the 32-byte anchor plus metadata to the Move contract. Figure 1 shows this data path, and Table 2 lists where each artifact resides and why.

The Move contract does not verify post-quantum signatures; it uses Sui’s object-centric storage model [24] to track scheme governance, endpoint registration, and commitment events. The contract does not embed scheme-specific signature-verification logic or signature encodings; public keys are retained on-chain as opaque byte vectors indexed by scheme identifier. The commitment object itself contains neither public keys nor signatures; its storage cost is tied to the anchor and metadata, not to signature length. Public-key length affects gas at registration, key attachment, and when the endpoint object is rewritten; the residual dependence on registered public-key length is quantified in the Results section.

Signature verification is performed off-chain by the auditor, because it is computationally expensive and because the signature scheme may still change. For a full audit, the auditor reads the commitment object or event, downloads the off-chain bundle from a content-addressed store such as Walrus [25], recomputes SHA-256(record) and compares it with the on-chain anchor, and finally verifies the endpoint signature.

The auditor CLI implements all of these steps except retrieval of the bundle from Walrus. Given --commitment-id, the CLI retrieves the named ProvenanceCommitment from the chain, checks that its anchor and scheme id match the bundle, then retrieves the Endpoint it belongs to and resolves the registered public key for that scheme at the commitment timestamp using KeyHistory; legacy endpoints use an explicitly labeled current-key comparison. Without --commitment-id, the CLI verifies only the bundle’s internal consistency: the record hash equals the anchor declared in the bundle, and the signature verifies under the bundle’s public key.

Internal consistency alone is not enough: a forger can generate a record, compute its own anchor, and sign with a key of its choosing, and the three internal checks still pass. The chain-binding transcript archived with the artifact (Appendix B) demonstrates this forgery case on a live Sui localnet; the same forged bundle is rejected once --commitment-id is supplied and the key is compared with the registered on-chain public key, while a genuine bundle in the same experiment verifies.

### 3.2. Threat Model and Design Goals

The adversary model follows the three classes defined for this endpoint class in prior work [11], narrowed here to the accountability objective of this paper. The class names are the same as in that work; the capabilities of each class are narrowed to the record layer, rather than to the theft of a design file or printing beyond authorization modeled there.

The first class is an **external attacker**. This adversary can reach the off-chain storage layer but holds no endpoint key and no capability. It may modify, replace, or backfill a stored record, and it may publish an alternative attestation bundle after an incident. Anchoring is aimed at this class: any edit breaks the recomputed digest, and the ledger timestamp anchors the record digest, although it does not timestamp the signature or the complete bundle.

The second class is a **malicious endpoint**. This adversary holds a valid key and capability and misuses them, for example, by claiming a decision it did not make, denying one it did, or attributing a decision to another device. The endpoint signature, together with the ownership and capability checks performed when a commitment is created, links a commitment to a key registered for the endpoint at the commitment’s ledger time, subject to the key-protection and lifecycle assumptions stated in Section 6. This class is therefore conditionally attributable rather than prevented.

The third class is an **authorized-but-curious endpoint**. This adversary passes every authorization check, legitimately obtains the material it needs to execute, and then acts in ways the authorization protocol can neither observe nor constrain; in the setting of [11], by retaining the decrypted design file for unauthorized secondary manufacturing. Prior work already identified this class as lying beyond the authorization layer. It is also a class that drives the present design, and this paper does not prevent that behavior either.

What the proposed framework changes is the evidential footprint the behavior leaves: every decision taken inside the sanctioned workflow carries a tamper-evident, ledger-anchored, endpoint-signed record, so work performed outside that workflow is distinguished not by detection at the time but by the absence of a corresponding record at audit. Establishing that absence requires linking physical items to the commitments expected for them, which lies outside the scope of this paper. The framework contribution to this class is therefore narrow but concrete: it preserves endpoint statements for investigation, subject to the key-protection and ledger-trust assumptions below.

The framework assumes that the blockchain provides append-only ordering for commitment transactions and that auditors can obtain candidate off-chain bundles. It assumes endpoint private keys are protected well enough for the evaluation period. It does not assume that firmware-reported telemetry is a physical ground truth, and it does not prevent a compromised endpoint from faithfully signing false telemetry. Table 3 summarizes the resulting design goals, the mechanisms that realize them, and their explicit boundaries.

## 4. Materials and Methods

### 4.1. Provenance Record and Audit Protocol

The prototype uses a structured record rather than an opaque log string. A record contains a schema version, a task identifier, an endpoint identifier, a decision label, a design-file hash, a sensor-log hash, a timestamp, a telemetry sample vector, and an optional previous anchor. The previous-anchor field can be used to string repeated decisions or print phases into a local hash chain, but the on-chain contract does not require that chain structure.

A deterministic serialization rule is essential. If the endpoint, auditor, and tests serialize fields differently, the same semantic record will produce different anchors. The prototype serializes the record with serde_json over a fixed struct field order, which is deterministic for a given schema version and toolchain; schema_version is the first serialized field, so it participates in the anchor. A production deployment should switch to a unique standardized encoding (for example, BCS or JCS), so that the hash no longer depends on the serializer in use. The Rust implementation treats anchor generation as a library concern and includes tests for anchor stability and bundle verification.

The audit protocol is simple enough to be reproduced independently. First, the auditor obtains the on-chain commitment for a target endpoint and time window. Second, the auditor retrieves the off-chain bundle indicated by the blob id. Third, the auditor recomputes the record hash and compares it with the commitment anchor. Fourth, the auditor verifies that the signature is valid for the record bytes, public key, and declared scheme. Finally, the auditor checks that the endpoint registration and scheme metadata match the expected device inventory. Appendix A traces this procedure through a disputed manufacturing job, and Table A1 maps each audit question to the bundle and on-chain evidence that answers it.

The five steps above can be written as an anchoring and an acceptance condition. Let *r* be a provenance record. The record bytes and the anchor are

(1) $$c(r)=\mathrm{serde}\_\mathrm{json}(r),\;a(r)=\mathrm{SHA}\text{-}256\left(c(r)\right).$$

c(r) is serialized in struct field order and is deterministic under this prototype’s toolchain; a(r) is fixed at 32 bytes. Let the attestation bundle be $B=(r,{\mathrm{sid}}_{B},pk_{B},\sigma_{B},a_{B})$, where sid denotes the one-byte signature-scheme identifier and σ the signature; let *C* be the on-chain commitment and *E* the endpoint. Audit accepts if and only if

(2) $$\begin{array}{cc} \mathrm{AuditAccept}(B,C,E,H) & \;\equiv a\left(r\right)=a_{B}=a_{C}\\ \; & \;\wedge \;{\mathrm{sid}}_{B}={\mathrm{sid}}_{C}\\ \; & \;\wedge \;pk^{*}\neq ⊥\;\wedge \;pk_{B}=pk^{*}\\ \; & \;\wedge \;{\mathrm{Verify}}_{{\mathrm{sid}}_{C}}\left(pk_{B},\;c\left(r\right),\;\sigma_{B}\right)=1. \end{array}$$

Here, *H* is the key history attached to *E*, and $pk^{*}=\mathrm{Resolve}\left(H,{\mathrm{sid}}_{C},C.\mathrm{created}\_\mathrm{ms}\right)$ is the public key whose interval contains the commitment timestamp; ⊥ denotes no matching key. An interval begins at added_ms and ends strictly before retired_ms, with zero retirement time denoting an open interval. Thus, the four conditions are anchor equality, scheme equality, existence and equality of the historically registered key, and signature validity. This formulation assumes that the auditor obtains *C* from the intended ledger and deployment and follows C.endpoint_id to the expected endpoint *E*, with *H* attached to that endpoint. Device inventory and deployment identity are external trust inputs: the CLI uses its configured Sui network and checks object-type suffixes, but does not independently pin a trusted package address. Legacy endpoints lacking *H* use a separately labeled current-key comparison and do not establish historical key validity. Omitting the on-chain comparisons establishes only bundle-internal consistency.

Three different counts appear in this paper because three different objects are being described, not because the procedure is stated inconsistently. Table 4 sets them side by side. The distinction matters for the reason just given: the three offline checks accept a bundle a forger can construct, and only the comparison against the on-chain commitment and the registered key rules it out. The fields of the provenance record itself, and the audit role of each, are listed in Table 5.

### 4.2. Move Contract and Scheme Governance

The Endpoint object of this package descends from the manufacturing_endpoint module of prior work [11] (one of seven Move modules there), which registered endpoints, mounted tasks, and verified authorization at execution time on behalf of the key server. What is new here is the governance and scheme-agility machinery built around it: a shared registry that issues one-byte scheme ids, a set of per-scheme public keys on the endpoint in place of a single key, a three-state scheme lifecycle, an optional per-endpoint commitment policy attached as a dynamic object field, and the fixed-shape provenance commitment itself.

The package defines a governance layer and a set of per-endpoint objects. At the core of the governance layer is the shared object SchemeRegistry: any transaction may read it, while writes are gated by the capability object RegistryAdminCap, which is required to add a scheme or change its lifecycle. The registry maps a one-byte scheme id to a SchemeDescriptor that records the scheme name, NIST security category (0 for classical schemes, 1–5 for post-quantum categories), a quantum-resistance flag, a maximum signature size for off-chain sanity checking, and lifecycle status. Scheme ids are issued sequentially by the registry and never reused, so an off-chain catalog can mirror the chain deterministically.

Per-endpoint objects are organized around Endpoint. Endpoint records the owner address, a label, and an active flag, and it maintains a table of public keys keyed by scheme id, so one device can hold several scheme keys at once. EndpointCap records the endpoint id and is transferred to the owner at registration. Each commitment produces a ProvenanceCommitment whose contents are fixed: endpoint id, anchor, blob id, record-schema label, scheme id, and ledger timestamp.

The endpoint’s key table records only the keys it currently holds, which is what the commitment path needs and all it needs. The keys it has held over time are kept separately, in a retained KeyHistory attached to the endpoint as a second dynamic object field. Each entry records the scheme id, the public key, the ledger time the key was attached, the ledger time it was detached, and whether detachment was a routine retirement or a compromise report. Registration opens the history with the endpoint’s first key; attaching a key appends an entry; detaching one closes the open entry for that scheme rather than deleting it. Placing the history in a dynamic object field is not incidental. A dynamic object field is a separate child object, so it enlarges neither the endpoint object nor the set of objects a commitment transaction touches, and create_commitment never borrows it. Registration and key rotation write the history and the auditor reads it when resolving a key by time; what no path does is borrow it during create_commitment. Section 5.4 reports what that separation is worth in measured gas.

**Figure 2:**
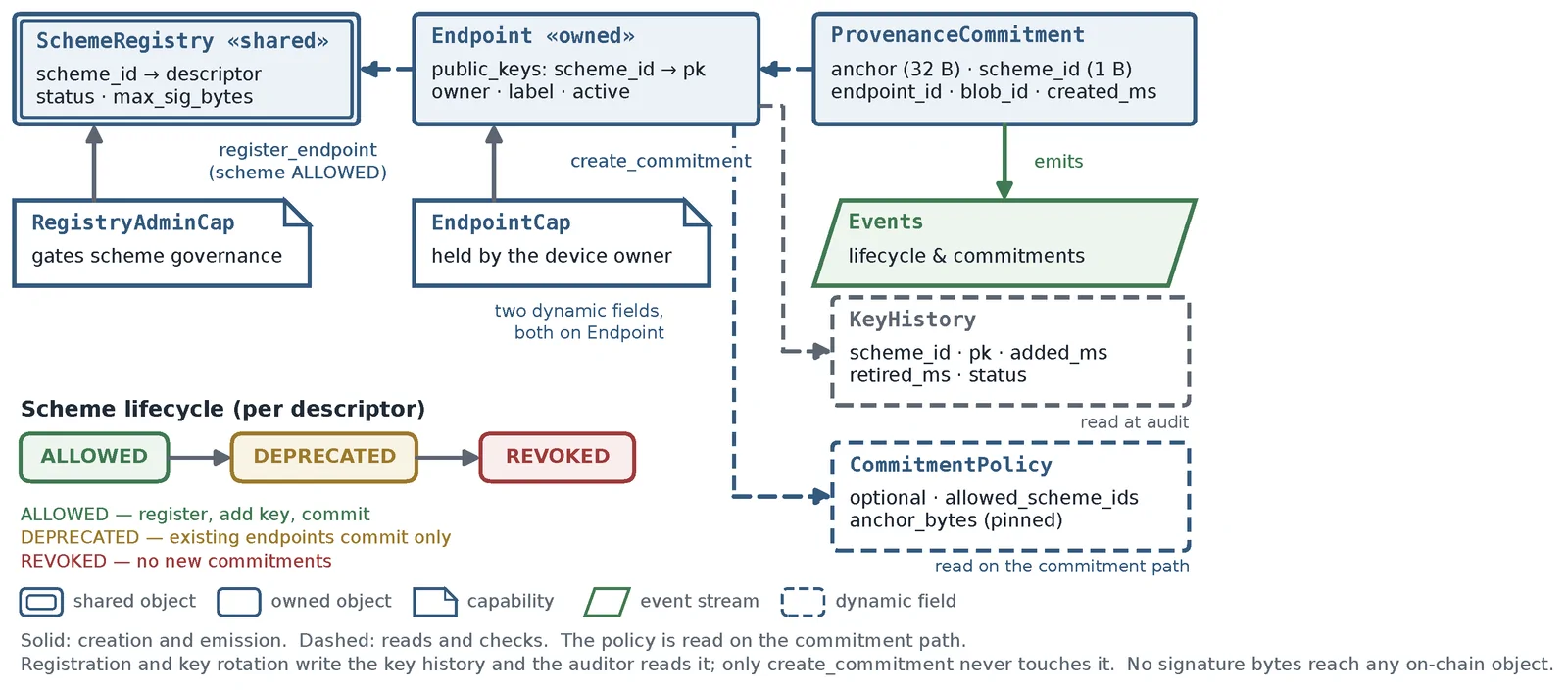
On-chain object model of the Move package. The shared SchemeRegistry issues sequential one-byte scheme ids and tracks a three-state lifecycle (ALLOWED, DEPRECATED, REVOKED); an Endpoint holds one public key per scheme id together with its owner capability (EndpointCap); two dynamic object fields hang off the endpoint independently of one another, an optional CommitmentPolicy and a retained KeyHistory, and neither is reached through the other. They differ in when they are touched, which is why line color separates them: the policy is consulted on the commitment path, while the key history is written by registration and key rotation and read at audit. Only create_commitment never touches it. Each commitment creates one fixed-shape ProvenanceCommitment and emits an indexable event. No signature bytes reach any on-chain object.

The policy layer is an optional CommitmentPolicy, attached to the endpoint as a dynamic object field. A policy can restrict commitments to an enumerated set of scheme ids, require a minimum NIST category, require a quantum-resistant scheme, and pin the anchor length. When no policy is attached, every scheme for which the endpoint already holds a key may be used, subject to the remaining checks, such as the registry not having revoked the scheme. Figure 2 summarizes this object graph and the scheme lifecycle.

Historical entries are retained, but their fields are not immutable: retirement updates an existing entry’s end time and status. The prototype allows at most 64 records across all schemes per endpoint; once that limit is reached, further key additions are rejected. History compaction and endpoint migration after exhaustion are outside this implementation.

Table 6 lists every entry function of the package and the checks it enforces. The register_endpoint entry function creates an endpoint and capability after the following checks pass: the requested scheme id exists in the registry and is in ALLOWED status (deprecated and revoked schemes are both refused for new registrations); the label is within 128 bytes; and the public key is non-empty and at most 4096 bytes. After registration, add_scheme_key and remove_scheme_key let the owner attach or detach a key for another scheme without replacing the endpoint object. Scheme migration and hybrid operation are expressed in this way: endpoint identity and commitment history survive a migration. The set_endpoint_status function lets the owner activate or deactivate the endpoint.

The create_commitment entry function applies a five-step check chain. First, the capability matches the endpoint, the sender is its owner, and the endpoint is active. Second, the endpoint holds a public key for the submitted scheme id. Third, that scheme id is registered and its registry status is not REVOKED. Fourth, any attached policy admits the scheme on all of its predicates. Fifth, the anchor is exactly 32 bytes. After the five steps, the blob id and record-schema strings are checked to be non-empty and within their length bounds. Thirty-eight Move unit tests cover the successful commitment path, registry governance, scheme migration and hybrid commitment, policy enforcement, and rejection of a mismatched capability, a wrong anchor length, a scheme the endpoint does not hold, a revoked scheme, and an inactive endpoint.

The commitment object contains no signature. Let the anchor be *a* and the scheme identifier sid. Then,

(3) C=(id,endpoint_id,a,blob_id,record_schema,sid,created_ms),|a|=32.

When a policy *P* is attached, write $D_{\mathrm{sid}}$ for the scheme’s descriptor in the registry:

(4) $$\begin{array}{cc} \mathrm{PolicyOK}(P,\mathrm{sid},a,D_{\mathrm{sid}}) & \;\equiv \left(P.\mathrm{allowed}\_\mathrm{scheme}\_\mathrm{ids}=⌀\vee \mathrm{sid}\in P.\mathrm{allowed}\_\mathrm{scheme}\_\mathrm{ids}\right)\\ \; & \;\wedge \;D_{\mathrm{sid}}.\mathrm{nist}\_\mathrm{level}\geq P.\min\_\mathrm{nist}\_\mathrm{level}\\ \; & \;\wedge \;\left(\neg P.\mathrm{require}\_\mathrm{quantum}\_\mathrm{resistant}\vee D_{\mathrm{sid}}.\mathrm{quantum}\_\mathrm{resistant}\right)\\ \; & \;\wedge \;|a|=P.\mathrm{anchor}\_\mathrm{bytes}. \end{array}$$

If no policy is attached, (4) is treated as passing. The core authorization and anchor checks of create_commitment pass if and only if

(5) $$\begin{array}{c} \;\mathrm{CreateOK}(E,\mathrm{cap},R,\mathrm{sid},a,\mathrm{sender})\\ \begin{array}{cc} \; & \equiv (\mathrm{sender}=E.\mathrm{owner})\wedge (\mathrm{cap}.\mathrm{endpoint}\_\mathrm{id}=\mathrm{id}(E))\wedge E.\mathrm{active}\\ \; & \wedge \;\mathrm{sid}\in \mathrm{dom}(E.\mathrm{public}\_\mathrm{keys})\\ \; & \wedge \;\mathrm{sid}\in \mathrm{dom}(R.\mathrm{schemes})\wedge R.\mathrm{schemes}[\mathrm{sid}].\mathrm{status}\neq \mathrm{REVOKED}\\ \; & \wedge \;\left(\neg \mathrm{HasPolicy}\left(E\right)\vee \mathrm{PolicyOK}(P_{E},\mathrm{sid},a,R.\mathrm{schemes}\left[\mathrm{sid}\right])\right)\\ \; & \wedge \;|a|=32. \end{array} \end{array}$$

**Figure 3:**
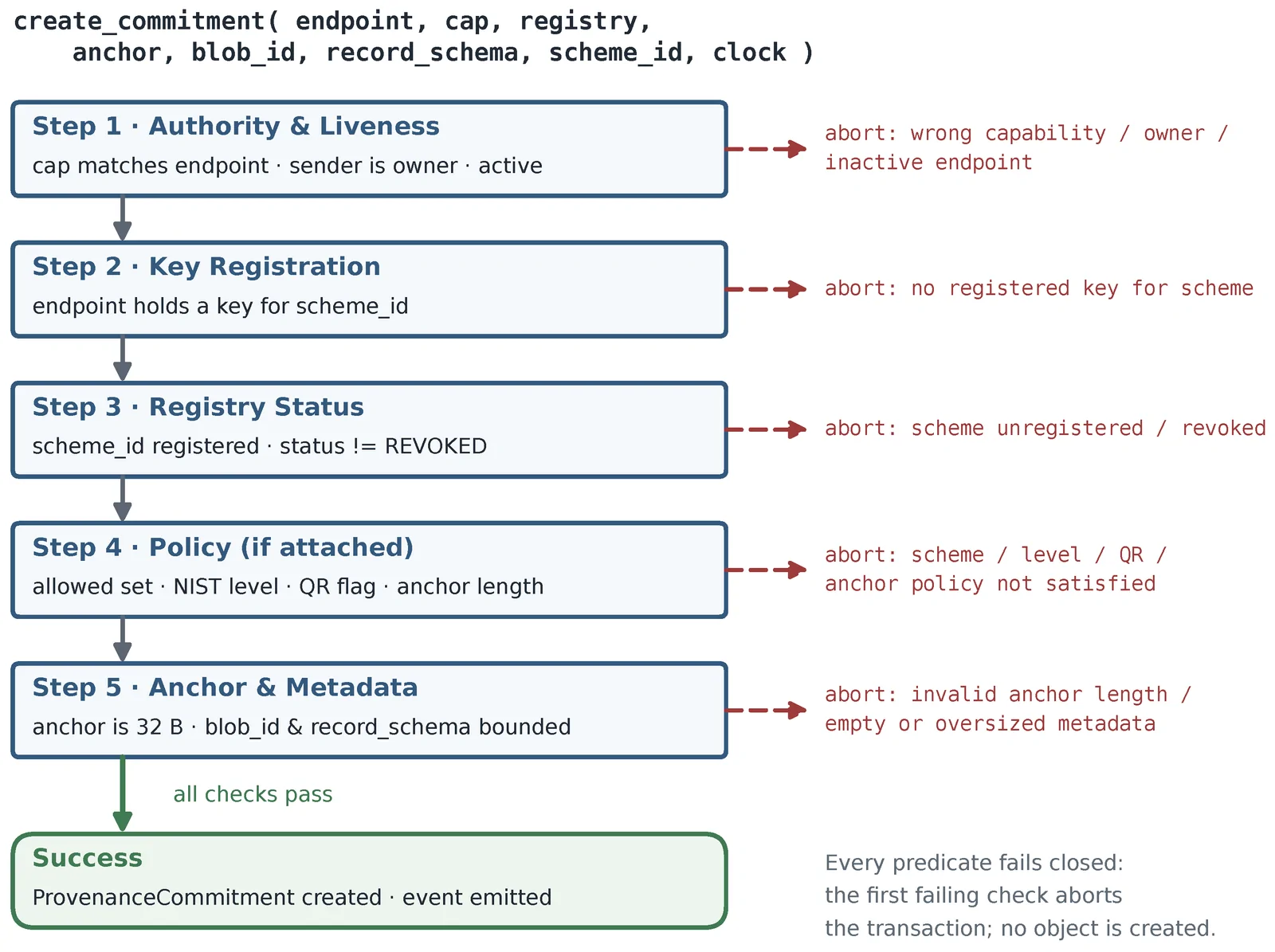
The five-step check chain of create_commitment and the abort conditions enforced at each step. Checks fail closed: the first failing check aborts the transaction and no object is created. Deprecation is not checked here: a deprecated scheme continues to serve existing endpoints, and only a revoked scheme blocks new commitments.

A deprecated scheme still lets existing endpoints create commitments; a revoked scheme does not. Length checks on blob_id and record_schema come after the five steps and are not part of (5). Figure 3 traces this check chain and the abort condition each step enforces.

These checks exist so that the object graph and metadata remain coherent enough for off-chain audit; the contract itself does not verify post-quantum signatures on-chain. Because a scheme can be referenced only by an id the registry has issued, a mistyped scheme name in an off-chain catalog cannot produce an endpoint that never matches any verifier. The contract emits nine event types, covering scheme registration and status change, endpoint registration, key addition and removal, endpoint status change, policy attachment and detachment, and provenance commitment, so indexers can build audit views without scanning every object individually.

### 4.3. Rust Endpoint and Auditor Implementation

Off-chain code is implemented in Rust. It constructs provenance records, computes anchors, and signs and verifies attestation bundles. The provenance module defines the telemetry and record data structures; the record is serialized and hashed with SHA-256 to obtain a 32-byte anchor. On verification, the module consults a scheme catalog that mirrors the on-chain registry ids and checks the bundle under Ed25519, ML-DSA, or SLH-DSA.

The command-line tool provenance-audit provides three subcommands: sample writes a bundle for one of the six catalog schemes, verify checks a stored bundle, and batch-verify repeats verification to measure throughput. Verify performs three checks and fails closed on each: the scheme id must resolve to a scheme name in the catalog; the recomputed SHA-256 must equal the anchor carried in the bundle; and the signature must verify under that scheme. If the name and id disagree, the bundle is rejected before signature verification. All three checks run locally; they do not query any on-chain object. A bundle is a seven-field JSON document: the record, the scheme name, the scheme id, the hex-encoded public key, the signature, the anchor, and an optional Walrus blob id. A scheme name that is not in the catalog is refused at construction time. The public artifact exposes a CLI rather than a demonstration UI, so that reviewers can replay the commands and benchmarks from transcripts without operating an application screen.

The benchmark CLI signs the same 638-byte record with each supported scheme, reports public-key and signature sizes together with key-generation, signing, and verification latency, emits both a Markdown table and CSV, and attaches an environment header with CPU, core count, governor, toolchain, and commit. The same binary is used for desktop development and for edge evaluation on a Raspberry Pi 5; the results appear in Section 5.

A note should be made about the signing-latency methodology. The libraries used here sign deterministically, and ML-DSA internally uses rejection sampling, so the number of retries for a given key and message is fixed. Signing the same key repeatedly therefore yields almost the same number each time: in an early run, the within-run standard deviation was about 4 us, while the median moved among 241, 416, and 931 us across three runs. That behavior is specific to deterministic signing; the hedged mode of FIPS 204 injects fresh randomness into every signature, so repetition would sample the retry distribution. The benchmark therefore generates a new key for each timed signature before timing starts. The statistic is signing time over different keys; key generation itself is excluded. Verification is measured by repeating one key and one signature per scheme; this measures a fixed-bundle workload rather than a distribution over keys or signatures.

### 4.4. Evaluation Methodology

The evaluation comprises three experiment groups. The first is the cryptographic microbenchmark implemented by pqc-bench, covering Ed25519, ML-DSA-44, ML-DSA-65, ML-DSA-87, SLH-DSA-SHA2-128f, and SLH-DSA-SHA2-128s, and measuring key-generation, signing, and verification latency, together with public-key and signature size. The second records on-chain gas and transaction effects for package publication and for the entry functions that seed the scheme registry, register endpoints, attach scheme keys, update endpoint status, and create commitments. The third exercises repeated bundle verification as an implementation microbenchmark. Because the current measurement repeats one archived ML-DSA-44 bundle, it is not treated as an end-to-end audit-throughput result. Table 7 summarizes the measurements, their metrics, and their purpose.

The microbenchmark was run on a Raspberry Pi 5 under the same Rust release profile, with operating-system version and CPU governor recorded. There were three runs of 200 iterations each; each timed block discarded a warm-up iteration, scheme order and the order of operations within a scheme were shuffled, and raw output was archived in full. Gas was measured on a Sui localnet; the throughput benchmark ran on the development desktop. Desktop microbenchmark results are retained as a same-methodology comparison, not as the edge-hardware evidence. The hardware platform matches prior work [11]: the manufacturing endpoint of that paper ran on the same board while driving a desktop 3D printer.

The main independent variables are signature scheme, record size, batch size, hardware platform, and Sui execution environment. The main dependent variables are signing latency, verification latency, signature size, total off-chain bundle size, on-chain transaction gas, and audit throughput. The 32-byte anchor is a fact of the object layout, not an experimental hypothesis. What the experiments confirm is whether on-chain commitment gas stays the same when the scheme or the record grows; signing latency per scheme on the Raspberry Pi 5; and the heavy tail of ML-DSA signing latency, measured as the 99th percentile relative to the median.

### 4.5. Crossed Key-by-Message Experiment

Table 8 establishes substantial dispersion in ML-DSA signing latency. A crossed design addresses a narrower question: whether a persistent key factor is detectable across messages in this implementation. This question is separate from whether selecting keys would improve latency on unseen messages, which would require an independent selection-and-evaluation experiment.

FIPS 204 and the Dilithium specification argue that the expected number of rejection iterations does not depend on the secret key, and they use that argument to conclude that counting iterations leaks nothing about the key. The argument is exact for the first rejection condition (the infinity norm of z). For the second condition (the low-order bits of w − cs2), the specification assumes heuristically that those bits are uniformly distributed. IETF guidance for constrained devices lists private-key material as one of four determinants of the rejection count, and notes that some message and key combinations iterate more than others [21]; that statement is analytic and does not conflict with expected value being independent of the private key, provided there is no systematic difference between keys. We did not identify a published crossed-design measurement that separated these two interpretations, so we ran one: what is tested here is attributable variance across keys, not the cost of a single (key, message) pair.

The design is a crossed grid: 40 freshly generated keys each sign the same 160 distinct 638-byte messages, with one timed signature per cell (6400 per scheme per replicate). Six replicates were collected on the Raspberry Pi 5 and six in each of two workstation sessions. Within a session, run-index-specific seeds generate different messages and execution orders; corresponding run indices across the three sessions reuse those seeds and hence the message sets and shuffled orders. These are 18 runs with fresh system-generated keys, not 18 independent message-set draws. The keys were not retained, so the same anomalous key sets cannot be remeasured. The deterministic signing path uses fixed signing randomness, and the program confirms byte-identical signatures by re-signing one cell per scheme, not the entire grid. This checks deterministic output, not deterministic elapsed time. Ed25519, which has no rejection sampling, provides a control for comparison without defining a universal measurement floor. Shuffled execution order reduces association between drift and key identity; per-scheme latency/order correlations are reported as diagnostics, not as proof that all timing confounds are absent.

For latencies $y_{km}$, we use the crossed random-effects description $y_{km}=\mu +u_{k}+v_{m}+e_{km}$. With one observation per cell, $e_{km}$ combines key-by-message interaction and measurement noise. Let ${\bar{y}}_{k\cdot }$, ${\bar{y}}_{\cdot m}$, and $\bar{y}$ denote the key, message, and grand means. The analysis computes

$$\begin{array}{cc} MS_{\mathrm{key}} & =\frac{M\sum_{k}{\left({\bar{y}}_{k\cdot }-\bar{y}\right)}^{2}}{K-1},\;MS_{\mathrm{msg}}=\frac{K\sum_{m}{\left({\bar{y}}_{\cdot m}-\bar{y}\right)}^{2}}{M-1},\\ MS_{\mathrm{res}} & =\frac{\sum_{k,m}{\left(y_{km}-{\bar{y}}_{k\cdot }-{\bar{y}}_{\cdot m}+\bar{y}\right)}^{2}}{\left(K-1)(M-1\right)},\\ {\hat{\sigma }}_{\mathrm{key}}^{2} & =\max\{\left(MS_{\mathrm{key}}-MS_{\mathrm{res}}\right)/M,0\},\\ {\hat{\sigma }}_{\mathrm{msg}}^{2} & =\max\{\left(MS_{\mathrm{msg}}-MS_{\mathrm{res}}\right)/K,0\}. \end{array}$$

The reported effect estimate is

$${\hat{\mathrm{ICC}}}_{\mathrm{key}}=\frac{{\hat{\sigma }}_{\mathrm{key}}^{2}}{{\hat{\sigma }}_{\mathrm{key}}^{2}+{\hat{\sigma }}_{\mathrm{msg}}^{2}+MS_{\mathrm{res}}}.$$

Negative component estimates are truncated at zero; an estimated zero is therefore not evidence of exact independence. The primary test shuffles key labels independently within each message and compares the untruncated key sum of squares over 10,000 permutations. It reports p=(b+1)/(10,000+1), where *b* counts permuted statistics at least as large as observed. Exchangeability under the null is required; the procedure avoids a normal-error assumption but does not remove implementation or temporal confounding. All archived tests use the seed label 20260810:scheme:key; the manuscript reports those archived Monte Carlo *p* values.

For traceability, the protocol’s planning reference was

(6) $$q_{K,M}=\frac{F_{0.95}\left(K-1,(K-1)(M-1)\right)-1}{M},$$

which is 0.002508 for K=40, M=160. Under a balanced homoscedastic random-effects model, $E\left[MS_{\mathrm{key}}\right]/E\left[MS_{\mathrm{res}}\right]=1+M\sigma_{\mathrm{key}}^{2}/\sigma_{\mathrm{res}}^{2}$. This is a ratio of expected mean squares, not generally E[F], and approximates an ICC-based reference only when the other components are sufficiently small. Equation (6) is retained only as a planning reference under the stated balanced random-effects approximation. It is not interpreted as a detection limit, confidence bound, or power guarantee. Both dimensions affect this planning quantity: increasing *K* to 80 at M=160 lowers it to 0.001728. We do not report a numerical population-variance upper bound from this calculation.

We report every collected replicate and additionally give Bonferroni-adjusted values over the 72 key-factor tests (18 runs, four schemes). This correction does not require independence between tests. Cross-platform consistency is supplementary evidence; a non-significant Pi result cannot negate a desktop rejection.

## 5. Results

### 5.1. Edge Cryptographic Performance

**Figure 4:**
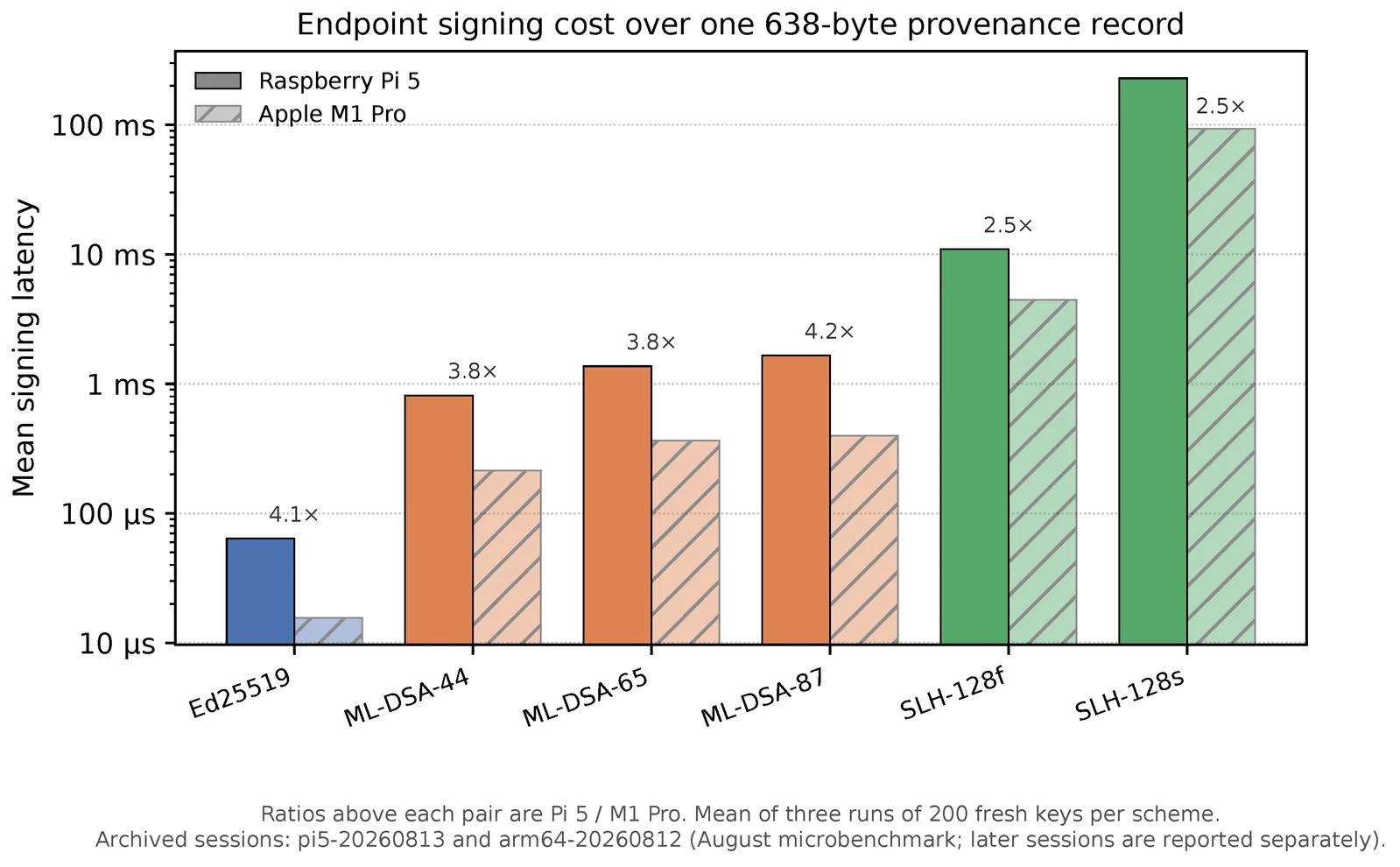
Signing latency per scheme on the Raspberry Pi 5 and the Apple M1 Pro development machine, log scale. On signing, the Pi 5 is 2.45 to 4.15 times slower depending on the scheme, but scheme order is preserved across platforms; SLH-DSA-SHA2-128s requires approximately 229 ms per signature on this platform and is therefore unsuitable for high-rate per-decision signing. Both platforms are the archived August microbenchmark sessions pi5-20260813 and arm64-20260812; the September sessions are separate measurements and are not pooled into this figure.

Table 8 reports three release-build runs of 200 iterations each on a Raspberry Pi 5 (Debian 13, four cores, 2.4 GHz) over the same 638-byte provenance record. The session ran with the CPU governor fixed at performance and the measurement pinned to a single core, and was verified free of thermal and under-voltage throttling throughout. A same-procedure run on the Apple M1 Pro development machine is archived alongside it; on signing, the Raspberry Pi 5 is 2.45 to 4.15 times slower depending on the scheme. Figure 4 plots the signing latencies of both platforms side by side: the scheme ordering from fastest to slowest is the same on both machines, so that ranking is not unique to a single platform.

The key-generation, signing, and verification columns of Table 8 are means of the per-run medians. The signing distribution is also given by its 95th and 99th percentiles and its coefficient of variation, because for rejection-sampled schemes a single figure does not describe signing latency. Percentiles are nearest-rank, so every value was measured rather than interpolated. For continuity with the archived summaries, the signing means in table order are 64.1, 814.3, 1368.3, 1656.0, 10,955.9, and 228,906.7 us.

Two cells do not reproduce within a factor of 1.10 across the three runs and should be read as approximate: ML-DSA-44 signing (763/594/588 us, 1.30×) and ML-DSA-87 signing (1208/1379/1201 us, 1.15×). Both schemes use rejection sampling, so a few signatures can be far slower than the median; sampling variability and host effects can move a median across runs. The coefficient of variation, which uses every sample rather than one order statistic, is far steadier: it spans 0.3 percentage points across the three runs for ML-DSA-44 and 1.7 for ML-DSA-87.

**Figure 5:**
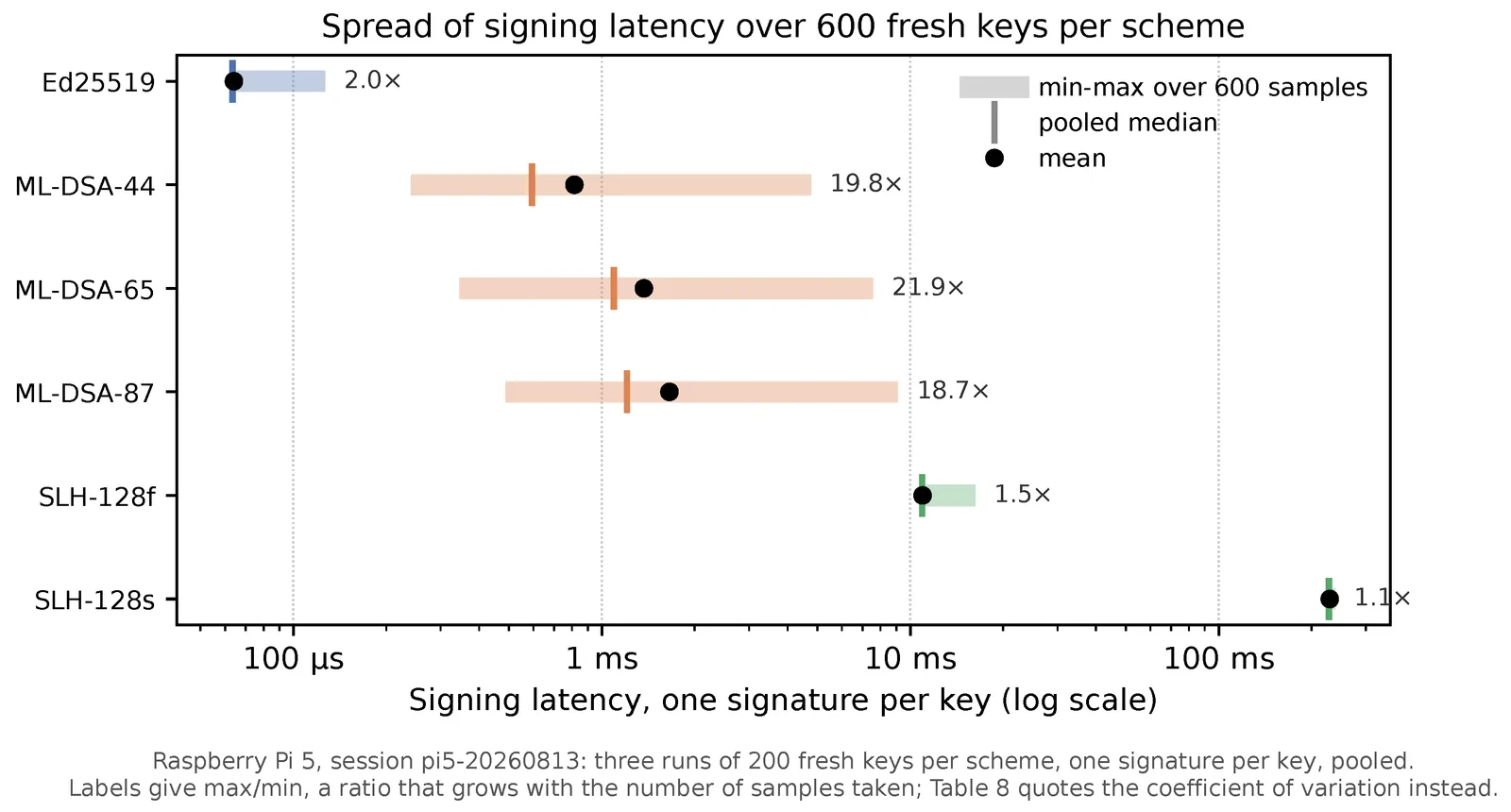
Per-key spread of signing latency on the Raspberry Pi 5, session pi5-20260813: min–max band, median, and mean over the pooled 600 samples, one signature per fresh key. The marked median is the median of the pooled samples (593.3 us for ML-DSA-44), not the 648.2 us column of Table 8, which is the mean of the three per-run medians. One sample per key means the band contains both key-to-key differences and per-invocation variation and does not separate them; quote the coefficient of variation in Table 8, because min–max band width grows with the number of samples taken.

These results support the central architectural decision, but they also expose a property that a single figure conceals. ML-DSA signatures are larger than Ed25519 signatures, yet at 648 us on a Raspberry Pi 5 (the mean of the per-run medians reported in Table 8; the pooled median marked in Figure 5 is 593 us), ML-DSA-44 remains plausible for decision-level events. ML-DSA signing uses rejection sampling, motivating a geometric retry model. Elapsed time is an indirect proxy for that mechanism; this microbenchmark does not separate persistent key differences from message interaction or host effects.

The consequence for a latency budget is direct. The mean of ML-DSA-44’s run-level 99th percentiles is 2852 us, about 4.4 times the mean of the three run medians (648 us). A threshold set to a true median has approximately 50 percent exceedance by definition; for reference, the pooled median of the 600 ML-DSA-44 samples is 593 us. For this measured workload, the empirical 99th percentile provides an approximately 1 percent exceedance threshold, although it is not a worst-case guarantee for future executions. Over three runs of 200 fresh keys each, the coefficient of variation of ML-DSA-44 signing latency was 78.1, 78.0, and 77.8 percent; ML-DSA-65 and ML-DSA-87 averaged 77.9 and 78.9 percent. SLH-DSA-SHA2-128s was at 0.1 percent, because it does not use rejection sampling. The coefficient of variation is more directly comparable than the sample range when implementations and sampling designs are sufficiently aligned. Under that qualification, the 78 to 79 percent measured here is higher than the 66.0 to 73.5 percent range reported for a Cortex-M0+ class device in [22]. A maximum-to-minimum ratio cannot be so compared, because the expected range of a sample grows with the number of samples taken. For completeness the per-run ML-DSA-44 ratios were 19.7, 17.5, and 14.0, and they should be read as a property of a 200-sample experiment rather than of the scheme.

Ed25519 at 6.5 percent CV and SLH-DSA-SHA2-128f at 2.1 percent show that measurable timing variation also occurs without rejection sampling. A small number of long observations can affect variance statistics strongly. Because no scheduler trace was collected, these samples cannot be attributed conclusively to interruptions. All samples are retained in the reported summaries.

**Figure 6:**
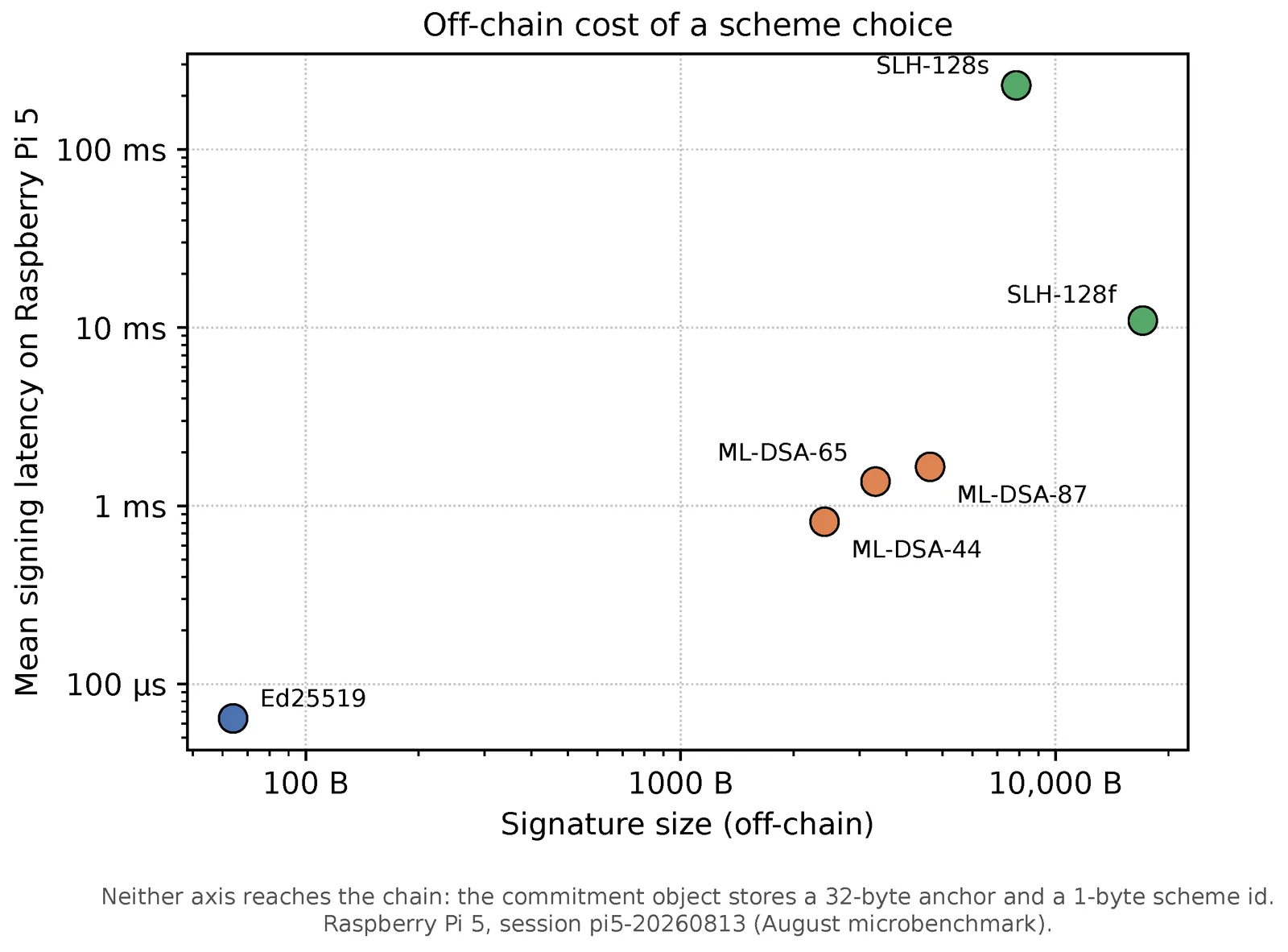
The off-chain tradeoff space on the Raspberry Pi 5: signature size against mean signing latency, log-log. Each scheme choice moves cost between the two off-chain axes; neither axis reaches the chain, which is the point of the anchor-only design. Session pi5-20260813, as in Figure 4.

Figure 5 makes the contrast visible: over 600 keys, the slowest ML-DSA-44 sample cost 19.8 times the fastest, consistent with the heavy-tailed behavior expected from rejection sampling, although host-level timing effects are not excluded; SLH-DSA stays within a factor of 1.5; Ed25519’s band also includes host-level timing variation. A device with a per-decision latency budget cannot be provisioned on the median alone; it must leave headroom for occasional slowdowns. SLH-DSA shows a different tradeoff, summarized in Figure 6: the public key is compact, but signature size and signing cost are much higher, and SLH-DSA-SHA2-128s, at approximately 229 ms per signature, is substantially less suitable for frequent decision-level signing on this platform.

Figure 4, Figure 5 and Figure 6 and Table 8 retain the August session as the primary microbenchmark, and the September runs are reported beside it rather than pooled into it. Each September session archived three further 200-iteration microbenchmark runs alongside the crossed experiment of Section 5.2; these are a repeat of the same fixed-record design, not additional replicates of the August session, and the two are kept separate because the desktop host’s operating system and load conditions differ between sessions. Across pi5-20260813 and pi5-20260912, ML-DSA-44’s pooled median signing latency was 593.3 and 593.9 us and its mean 814.3 and 842.0 us over 600 samples each, with no samples excluded. These are descriptive comparisons of two sessions, not a test of equal distributions.

### 5.2. Key-Attributable Variance in ML-DSA Signing

On the Pi 5, none of the 18 ML-DSA key-factor tests rejects at nominal α=0.05. The largest estimated ML-DSA ICC is 0.0018 (ML-DSA-44, run 01; p=0.108). Ed25519 reaches 0.0025 in run 06 (p=0.0427). These observations describe this dataset; neither the comparison control nor the planning reference in Equation (6) provides a confidence upper bound on an undetected component.

The workstation sessions contain two nominally significant ML-DSA-44 results: Desktop A run 04 has ICC 0.006987 and p=0.00029997; Desktop B run 02 has ICC 0.003304 and p=0.018698. Multiplying the archived *p* values by 72 and capping at 1 gives 0.02160 and 1.000, respectively. The first result therefore remains significant under this correction. Its mean is 214.36 us, with estimated key variance 208.99 us^2^, message variance truncated to zero, residual mean square 29,702.96 us^2^, and $F_{\mathrm{key}}=2.12575$. The corresponding key standard deviation is 14.46 us. Counting only 3 nominal rejections among 72 tests cannot dismiss the surviving result. The reported *p* values are finite-permutation estimates and should not be read with more precision than their Monte Carlo resolution supports.

The two excursions do not recur consistently across fresh key sets, and the original keys cannot be remeasured because they were not retained. We therefore report a desktop timing association with the key factor whose cause and persistence remain unresolved, rather than asserting either a cryptographic key effect or exact key independence. Absence of a Pi rejection is useful cross-platform evidence but is not a replication with the anomalous desktop keys.

Diagnostics narrow some explanations without excluding all measurement effects. In Desktop A run 04, the ML-DSA-44 latency/order correlation is −0.0119 and 2 of 6400 cells exceed ten times the replicate median. Such a threshold flags unusually long measurements; it does not identify scheduler preemption. The workstation sessions were not quiesced, and Table 9 also exposes larger drift in an Ed25519 run. A fitted geometric-retry model is a consistency check only: no rejection-loop counters were recorded, and its scale and overhead use observed mean and minimum latency. With one observation per cell, interaction and measurement noise are inseparable; structured noise can affect the estimated key component as well. Further work would retain benchmark-only keys, measure repeated cells and retry counters, and evaluate selected keys on held-out messages. The current study supports tail-aware latency budgeting but does not quantify the benefit or futility of key selection.

### 5.3. Off-Chain Evidence Size and Verification Microbenchmark

The cost that scheme choice does move is off-chain bundle size. The archived sample bundles are 1358 bytes for Ed25519, 8632 bytes for ML-DSA-44, and 35,416 bytes for SLH-DSA-SHA2-128f. These are hex-encoded JSON, so a binary encoding would remove most text-encoding overhead, whereas Base64 would reduce the expansion relative to hexadecimal encoding. The measured JSON sizes are therefore implementation-level storage figures; the relative differences between schemes remain the more general result and follow signature size. For an endpoint on a constrained uplink, this is the number that governs deployment, not the on-chain cost. The Move contract is insulated from this variation because it stores a 32-byte anchor and a one-byte scheme id, not the signature and not a variable-length scheme label. No per-decision signature bytes or variable-length signature field enter the create_commitment call or the ProvenanceCommitment object. Scheme choice can nevertheless affect other on-chain endpoint state through registered public-key length, as quantified in Section 5.4 and Section 5.6. This does not eliminate off-chain bandwidth and storage concerns, but it keeps the recurring commitment object’s serialized shape and size independent of signature length. In an IoT deployment this distinction matters because on-chain costs can be harder to change after deployment than off-chain record-retention policies.

Verification is cheaper than signing for the post-quantum schemes, which is useful for audit workflows: a third-party auditor may need to verify many bundles, while an endpoint signs only its own decisions. On the Raspberry Pi 5, ML-DSA-44 verification at 104 us is in fact faster than Ed25519 verification at 182 us, and the same ordering holds on the development machine. This ordering is a property of the two pure-Rust implementations listed in Table A2 at their default features, measured one signature at a time, on this study’s hardware, and not a general property of the algorithms. Batch-verification strategies were not evaluated and may change the ordering. It should be read as evidence that migrating to ML-DSA-44 does not penalize the auditor on this platform, not as a general ranking. A release-profile repeated-bundle microbenchmark over one archived ML-DSA-44 bundle completed 100 verification operations with no failures and reported 8,433.78 operations per second on the development machine. This repeated-bundle measurement is treated only as an implementation microbenchmark; audit-throughput claims require larger sets of distinct bundles, all supported schemes, and inclusion of storage and I/O costs.

### 5.4. On-Chain Commitment Cost Across Signature Schemes

Table 10 reports Sui localnet transaction effects captured on 11 September 2026 with Sui CLI 1.72.1 against the key-lifecycle package described in Section 4.2. The isolated session archives publication, registry setup, endpoint/key lifecycle operations, and commitments. The 13 August session and a September rerun provide comparisons before the lifecycle change. These are localnet measurements, not public-network or production prices.

**Figure 7:**
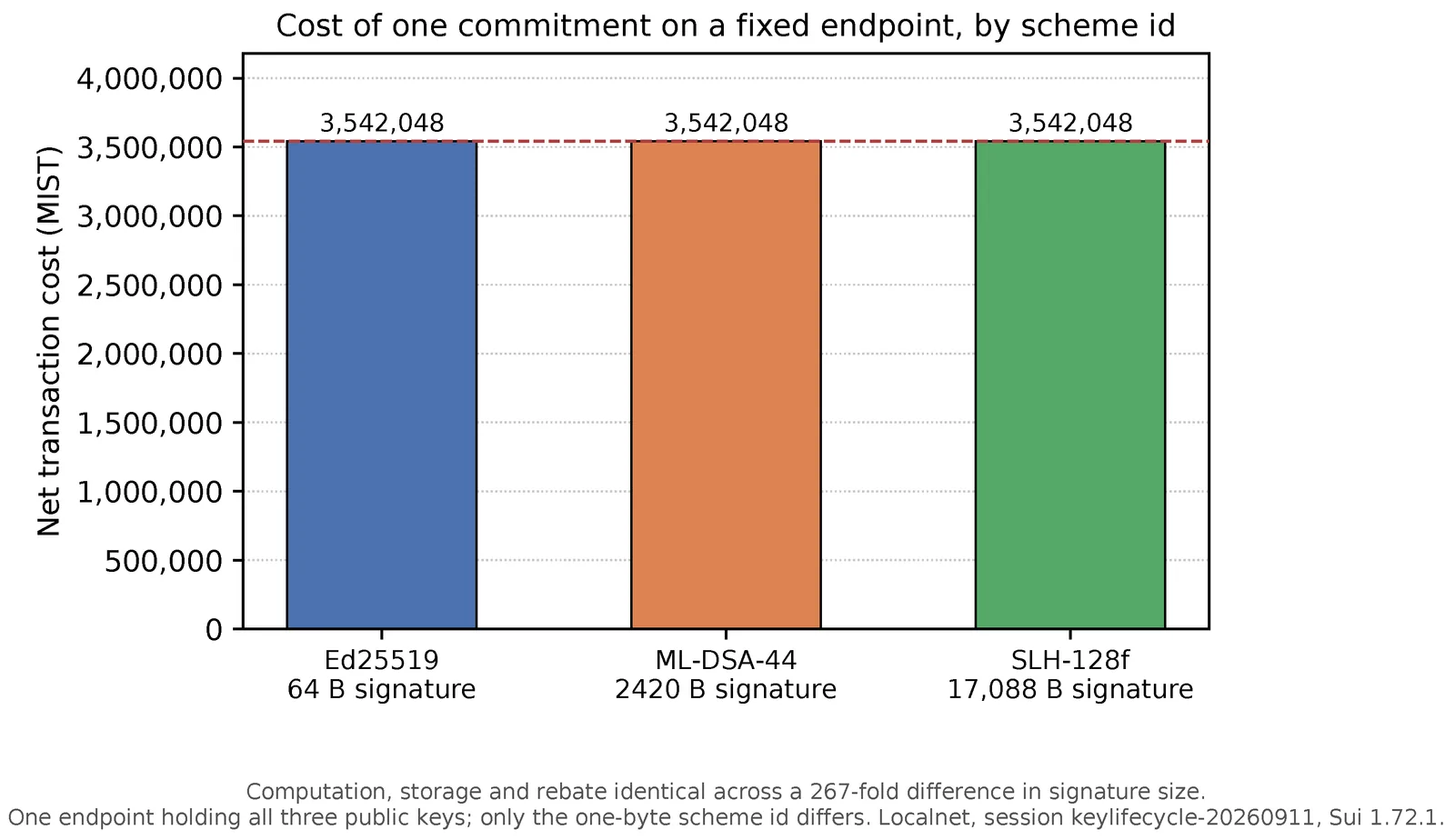
On-chain commitment gas is invariant across the evaluated schemes for a fixed multi-key endpoint. The same endpoint that already holds several scheme public keys committed under Ed25519, ML-DSA-44, and SLH-DSA-SHA2-128f, varying only the one-byte scheme-id argument; computation, storage and rebate were equal in every component, for a net transaction cost of 3,542,048 MIST, despite a 267-fold spread in off-chain signature size (64 B to 17,088 B). Measured on the 11 September lifecycle session keylifecycle-20260911, that is, against the contract that carries KeyHistory; localnet, Sui CLI 1.72.1. The dashed line marks the common net cost of 3,542,048 MIST shared by all three schemes.

The entry-function rows support the claim that the recurring commitment path is not sensitive to post-quantum signature byte length, and the registry design makes this property explicit in the object layout. Signature bytes are never passed into the Move call, and the commitment path stores a fixed 32-byte anchor, bounded metadata, and a one-byte scheme id. Repeating create_commitment against the same endpoint that already holds several scheme public keys, under Ed25519, ML-DSA-44, and SLH-DSA-SHA2-128f, varying only the scheme-id argument, produced identical measured gas components: 1,000,000 computation, 17,198,800 storage, 14,656,752 rebate, and 3,542,048 total MIST in all three cases, across a 267-fold difference in off-chain signature size. Figure 7 visualizes this measured cost invariance for a fixed endpoint state.

One qualification belongs with this result. Each commitment also rewrites the endpoint object and its capability, and Sui retains one percent of the previously charged storage cost on such rewrites, which is the 148,048 MIST component of the total above. The size of the endpoint object grows with the set of public keys it holds, so the per-commitment total carries a weak dependence on registered public-key length: on the multi-key endpoint measured here it is 3,542,048 MIST, while an endpoint holding only a 32-byte Ed25519 key would pay approximately three percent less and one holding only a 2592-byte ML-DSA-87 key approximately three percent more. The invariance reported above is therefore a statement about the fixed-shape commitment object, and about varying the scheme used for a given endpoint. It is not a statement that two endpoints provisioned with different key sets pay the same. This distinction matters because the dependence is on registered public-key state, whose cost includes registration and recurring rewrites, and not on per-decision signature length, which is what the 267-fold spread refers to and what never reaches the chain at all.

Adding the key-lifecycle record tests that separation a second way, because it enlarges endpoint state without enlarging the object the commitment path rewrites. The lifecycle record is held in a dynamic object field, so it is a child object that create_commitment never borrows. Re-measuring the whole session against the package that carries it reproduced every commitment figure exactly: 1,000,000 computation, 17,198,800 storage, 14,656,752 rebate and 3,542,048 total on the multi-key endpoint, and 3,439,980 on the single-scheme Ed25519 endpoint, with the transaction still creating one object and mutating three. The spread across single-scheme endpoints also stayed at 5.66 percent. What did move is the one-time path, as expected: registration roughly doubled (2.01 to 2.05 times, since the key is now recorded twice, once in the endpoint’s active set and once in its history), key attachment rose by 1.36 to 1.92 times, key removal is a new operation at 1,014,592 MIST, and publication rose by 1.25 times. Registry seeding and endpoint status changes were unchanged in their measured gas components, since neither touches the history. The separation the design rests on is therefore not an artifact of the feature set measured: state that the auditor needs but the commitment path does not read can be added at zero recurring cost.

Registration and key attachment populate both the active key set and KeyHistory. In the lifecycle session, attaching the 1,312-byte ML-DSA-44 key cost 21,182,560 MIST and attaching the 32-byte SLH-DSA-SHA2-128f key cost 1,912,532 MIST. These are different endpoint states and operation histories, so the figures should not be treated as a pure key-length slope. Recurring commitment cost retains the endpoint-rewrite dependence described above; publication is a separate deployment cost.

### 5.5. Record-Size Invariance and On-Chain Alternatives

The most important design choice is what not to put on-chain. A naive design would store the complete record and signature in a smart contract for maximum visibility. That approach is expensive, brittle, and tightly coupled to the chosen signature scheme. This design stores only enough to make tampering detectable, a 32-byte anchor and a one-byte scheme identifier, and leaves the full evidence bundle outside the ledger.

That choice is worth stating only if the alternative is priced, so we measured it on the same chain in the same session. In the evaluated localnet configuration, the observed marginal charge is 7600 MIST per additional serialized byte. The protocol configuration used for the run reports 100 storage gas units per byte (obj_data_cost_refundable) and a storage price of 76 MIST per gas unit (storage_price). This figure is a marginal storage term, not a fixed total transaction cost per byte. A stored object also carries an overhead independent of payload length, but store_anchor and store_payload are otherwise identical entry functions, so subtracting one from the other cancels that overhead and isolates the per-byte term. The storage rebate does not enter the extrapolation either: in these transactions the rebate is returned on the objects the transaction rewrites rather than on the object it creates, and it is accordingly identical (978,120 MIST) at every payload size measured. Under the evaluated rebate policy, later deletion can refund 99 percent of the stored object’s original storage charge; deletion transaction computation and other object effects still have to be paid. The retained storage component alone is 76 MIST per serialized byte, not an all-inclusive lifetime transaction cost. Against that line the anchoring path is a constant: on a single-scheme Ed25519 endpoint, committing a 634-byte record and a 2,092,961-byte record produced identical measured gas (3,439,980 MIST) across a 3301-fold range of record size, because in both cases the same 32-byte digest is what reaches the chain. The 3,542,048 MIST reported in Table 10 is the same operation on the multi-key hybrid endpoint, whose larger registered key set makes its endpoint-object rewrite more expensive.

Table 11 reports the subtraction that isolates the per-byte term, and Table 12 names every operation and baseline entered into the comparison, so that storing data and verifying a signature are priced separately rather than together.

The records behind those commitments are not hypothetical. Four of the six were built from a telemetry trace recorded on the Raspberry Pi 5 itself: 600 samples at 1 Hz of SoC temperature, clock frequency, throttling state, and load average, taken under synthetic CPU load, so the SoC warmed from 51 to 72.5 °C and the trace has the shape of a device doing work rather than a flat idle line. Truncating it at 3, 60, 300, and 600 samples gives records of 634, 5804, 27,850, and 55,450 bytes. The two largest records, at 344,174 and 2,092,961 bytes, are synthetic, because they correspond to one and six hours of sampling and no trace of that length was captured; they extend the range rather than evidence it, and the archived results label which is which.

This trace shows two things, and only the first is about cost. A ten-minute trace already produces a record two orders of magnitude larger than the 638-byte sample record used elsewhere in this paper, so record growth under real operation is not a marginal effect. And this trace is the only measurement reported here that reads a physical sensor. It is a genuine hardware measurement and it makes record growth real rather than assumed, but a Raspberry Pi warming under load is not manufacturing telemetry, and it does not turn this into a sensing study. The record schema retains the field names of its 3D-printing scenario, so in these records hotend_c carries a measured SoC temperature and fan_pct carries CPU load; the mapping is stated here because a field named for a printer while holding a processor reading would otherwise mislead.

The measured invariance to record size has two consequences. First, record size stops being a cost variable. An endpoint’s telemetry is unbounded: a task sampling once a second produces a record that grows without limit, while its anchor is 32 bytes however long the task runs. Second, large post-quantum signatures are not merely expensive on-chain but can be inadmissible: SLH-DSA-SHA2-128f’s 17,088-byte signature exceeds this chain’s 16,384-byte limit on a single transaction argument and is rejected before it reaches the chain at all. For that parameter set the straightforward on-chain-signature design is unavailable at any price, and a design would have to split the signature across arguments, objects, or transactions before verifying any of it.

We also measured where the design provides little benefit. For Ed25519 the saving is close to nothing: this chain verifies Ed25519 natively at 0.36 times the cost of storing an anchor, and verifying and retaining the 64-byte signature costs 1.09 times. The advantage is entirely a post-quantum effect, an order of magnitude for ML-DSA and a hard transaction-size constraint for SLH-DSA-SHA2-128f. Reporting a large classical saving would overstate the result, and the classical baseline is what establishes that the effect is driven by signature size rather than by the anchoring pattern itself.

### 5.6. Residual Dependence on Registered Public-Key State

The commitment object’s serialized shape and size are independent of signature length by construction. The total cost of the transaction is not quite independent of the endpoint’s registered public-key length: create_commitment also rewrites the Endpoint object it is called against, and that object carries the endpoint’s registered public keys. Measured on separate single-scheme endpoints, a commitment costs 2.83, 4.25, and 5.66 percent more for endpoints holding ML-DSA-44, ML-DSA-65, and ML-DSA-87 public keys than for one holding only Ed25519, while two endpoints holding different schemes with identically sized 32-byte keys differ by 0.01 percent. The dependence is on key size, not on scheme identity.

We report this because our own earlier experiment could not have detected it. All three of the original measurements were taken on a single multi-key endpoint holding every key and varying only the scheme_id argument; repeated here, that configuration again returns identical measured gas across three schemes. The earlier result was self-consistent and simply blind to this axis.

The distinction that matters for the claim is between the two lengths involved. The dependence is on *public-key* length, registered once per endpoint, and not on *signature* length, produced once per decision. The 267-fold difference in signature size that motivates this work still reaches the chain in no form at all.

## 6. Security Analysis

The framework prevents a simple record-replacement attack. If an adversary changes any field in the off-chain record, the recomputed SHA-256 digest will not match the on-chain anchor. If the adversary submits a different off-chain bundle with the same blob id but different content, the auditor will detect the mismatch. If the adversary creates a new record after the incident and signs it, the on-chain timestamp and event history reveal that the commitment was not present at the original time. Table 13 summarizes the attacks considered, their detection or mitigation, and the residual risk of each.

The framework also reduces algorithm-migration risk, and the scheme registry makes the migration path explicit rather than implicit. Historical commitments record a one-byte scheme id that resolves to a registry descriptor; they do not embed an on-chain verifier. An endpoint can be given a key for a new scheme and have its old key removed without replacing the endpoint object, so device identity and commitment history survive a migration, and holding two keys at once expresses a hybrid transition. Retiring a scheme fleet-wide becomes a single governed status change rather than a redeployment: a deprecated scheme continues to serve existing endpoints but accepts no new registrations or key attachments, while a revoked scheme is refused for new commitments outright.

Key rotation retains ledger-time validity windows for registered keys. The auditor resolves the key at the commitment timestamp, so removing an old key does not erase the association needed to audit an earlier commitment. The window is half-open: a commitment at the same ledger-millisecond as retirement or a compromise report is outside that old key’s window, even if transaction order places it earlier within the millisecond. Because the prototype records key-validity boundaries at millisecond resolution, transactions sharing the same ledger timestamp cannot be ordered solely from added_ms and retired_ms; the current implementation therefore applies this conservative half-open rule. A production deployment should additionally bind key-history transitions to a monotonic ledger-order identifier, such as a checkpoint or transaction sequence (or an equivalent chain-native ordering primitive). A replacement key has its own window. Legacy endpoints without history use an explicitly labeled current-key comparison. These are registered-key association rules, not proof of non-repudiation: a compromise report records when the owner reported compromise, not when the private key was actually lost. Attribution requires key protection during the relevant interval and trust in the intended ledger/deployment and endpoint inventory. Moreover, the anchor commits to record bytes, not the signature or complete bundle; a valid signature supplied later does not by itself prove that signature existed at commitment time. The ledger demonstrates anchoring by the authorized endpoint capability, while the signature demonstrates possession of the historically registered key when signing occurred.

The key limitation is endpoint truthfulness. If a compromised endpoint signs a false temperature value, the framework preserves that false claim rather than correcting it. Stronger physical assurance would require secure boot, trusted execution, remote attestation, calibrated sensor chains, or independent observers. The contribution here is tamper-evident accountability for endpoint statements, not an oracle for physical state.

A second limitation is blockchain-layer post-quantum security. The proposed PQC signature is an application-layer endpoint signature over the provenance record. The Sui transaction that creates the commitment still relies on the chain’s transaction authentication model. If the objective is end-to-end post-quantum assurance, the blockchain account and transaction layer must also evolve.

## 7. Discussion

### 7.1. Implications for WSN/IoT Secure Data Management

The results support a specific design principle for secure data management in WSN/IoT edge systems: high-volume or scheme-dependent evidence should not automatically become recurring ledger state. The blockchain is used here as an external ordering and integrity reference, while cryptographic verification and complete decision evidence remain at the edge/auditor layer. This division is particularly useful for sensing-to-actuation workflows because record size can grow with telemetry density and task duration, whereas the security value needed from the ledger is often only a stable commitment, device binding, and timestamped ordering point.

The results should not be read as an argument that blockchain is necessary for every WSN or IoT deployment. A trusted append-only service or another independently governed transparency mechanism could provide a similar ordering role. The benefit demonstrated here is narrower: when a ledger is already part of the security architecture, fixed-width anchoring prevents endpoint signature size and record volume from becoming recurring on-chain cost variables.

### 7.2. Generalizability and Ledger-Specific Effects

Every gas figure in this paper was measured on a single Sui localnet at the protocol version recorded in the archived artifact, and readers should treat them accordingly.

**What is ledger-specific and not transferable?** The 7600 MIST per serialized byte of marginal storage charge; the 16,384-byte maximum size of a single transaction argument; the availability of a native Ed25519 verification primitive, which many ledgers do not provide for this curve; and the residual dependence on endpoint key size described in Section 5.6, which arises from this chain charging for rewriting owned objects together with its non-refundable storage fee. Ledgers that do not charge for owned-object rewrites do not exhibit that dependence at all. The archived MIST-denominated components reproduce in the matched localnet setup; this does not fix costs under other protocol settings or the fiat value of SUI. That determinism was checked rather than assumed: the August session was re-run in full a month later, on the same CLI version and from the same isolated configuration, and every gas component of every transaction matched the archived effects exactly.

**What is structural and expected to hold on any ledger?** The independence of commitment cost from record size. Its cause is that the record never reaches the chain in any form, only a fixed-width digest of it, so the argument does not depend on how a particular ledger prices storage. Each ledger supplies its own constant and its own limits; none of them changes the fact that an SHA-256 digest is 32 bytes whatever it digests.

The *direction* of the post-quantum result should also generalize, even though its *magnitude* will not. Signature sizes between two and seventeen kilobytes are large relative to the per-transaction budgets of the ledgers we are aware of, so a design that keeps them off-chain avoids a real cost wherever it is deployed. Whether that cost appears as a price or as a hard limit, and how large it is, is a property of the target chain and has to be measured there rather than extrapolated from these figures.

### 7.3. Selecting an Endpoint Signature Scheme

The benchmark is most useful as a decision procedure rather than as a ranking. Which constraint binds first depends on the deployment, and the measurements identify the candidates.

**Decision rate sets the floor.** SLH-DSA-SHA2-128s signs in 229 ms on a Raspberry Pi 5, capping sustained decisions at roughly four per second on one core before signing alone saturates it. SLH-DSA-SHA2-128f at 11 ms, and ML-DSA at a median of 0.65 to 1.3 ms, leave correspondingly more headroom.

**Audit horizon decides whether the classical option survives.** Ed25519 has the lowest signing latency, the smallest signature, and the smallest evidence bundle among the evaluated schemes. ML-DSA-44 verifies faster in the measured Rust implementation, so Ed25519 is not lowest on every measured metric. It remains attractive where long-term post-quantum verification is not required; where such assurance is required, a post-quantum scheme is necessary.

**Bundle size decides transport and retention cost.** The full evidence bundles are 1,358 bytes for Ed25519, 8,632 bytes for ML-DSA-44, and 35,416 bytes for SLH-DSA-SHA2-128f in the hexadecimal encoding used by the artifact. A binary container would substantially reduce the hexadecimal expansion of binary fields, but it would not necessarily halve the entire bundle because non-binary record fields remain. For an endpoint on a metered link, this is often the constraint that binds before signing latency does.

**On-chain admissibility is a threshold rather than a cost.** If a deployment ever needs the signature itself on-chain rather than an anchor, a single 17,088-byte SLH-DSA-SHA2-128f argument exceeds the evaluated limit. Splitting the signature across arguments or objects is possible but was not benchmarked. Direct ML-DSA payload storage cost 7.51–13.53 times the storage-only anchor baseline for the two sizes in Table 12; this does not include post-quantum verification.

**Latency variance decides how a timing budget is sized rather than which scheme is chosen**; Section 7.4 treats it separately because it is the constraint most easily misread.

Applying these criteria to the endpoint class studied here, a Linux-class edge gateway making occasional actuation decisions that must remain auditable for years, ML-DSA-44 is the reasonable default: post-quantum, a signature small enough to transport and to admit on-chain if ever required, and a signing cost near one millisecond. SLH-DSA remains preferable where a conservative hash-based security argument is required and the decision rate is low enough to absorb its cost. Neither conclusion depends on the ledger; both depend on the endpoint.

### 7.4. Tail-Aware Scheduling for ML-DSA

ML-DSA signing is rejection-sampled: the signer produces a candidate, tests whether it falls within the required bounds, and discards it and retries when it does not. The number of attempts is random, so signing cost is a distribution over retries rather than a fixed duration, and a mean conceals the part of it that matters operationally.

On a Raspberry Pi 5 with the CPU governor fixed and the measurement pinned to one core, the three run-level ML-DSA-44 medians range from 588 to 763 us, the 95th percentiles from 1.87 to 2.19 ms, and the 99th percentiles from 2.50 to 3.03 ms. Thus, the run-level 99th percentiles are approximately four to five times the corresponding medians. A threshold set to a true median has approximately 50 percent exceedance by definition; for this measured workload, the empirical 99th percentile corresponds to approximately 1 percent exceedance and should be interpreted as a measured percentile rather than a worst-case deadline. Across the three ML-DSA parameter sets, the coefficients of variation in Table 8 average 78 to 79 percent; the per-run single values lie between 69 and 86 percent. SLH-DSA is about 1 percent; it does not use rejection sampling and is therefore slow but predictable.

The practical consequence is a scheduling one. An endpoint that must sign a decision before actuating, and that sizes its timing budget on the mean, will exceed that budget on the tail of the distribution. On a device driving physical equipment a missed deadline is a failure rather than a slow response, so a percentile chosen for the application’s tolerated exceedance rate is more informative than a median-only budget. An empirical 99th percentile targets roughly 1 percent exceedance in the measured workload; it is not a worst-case deadline guarantee for future executions.

The crossed experiment in Section 5.2 does not establish how key selection performs on future messages. Choosing the fastest observed key and evaluating its tail on the same measurements introduces selection bias; optimizing a sample mean also does not optimize the 99th percentile. We therefore make no quantitative key-selection claim. The scheduling recommendation follows directly from the observed microbenchmark tail, irrespective of the unresolved source of the two desktop key-factor excursions.

Ed25519 and SLH-DSA do not use rejection sampling, but elapsed-time measurements on a general-purpose operating system can still vary. A long sample alone cannot establish whether an interruption occurred; no scheduler trace was collected. This is a reason to state the measurement environment and report distribution summaries, not to assign all non-rejection-sampled variation to the operating system.

Two cautions apply to how this distribution is characterized. First, the coefficient of variation is more comparable than the sample range when implementations, workloads, and sampling designs are sufficiently aligned. The expected range of a sample grows with the number of samples taken, so a maximum-to-minimum ratio describes the size of the experiment as much as the behavior of the scheme and should not be compared directly across studies. Second, a long-tailed latency measurement on a general-purpose operating system invites a competing explanation, because a scheduler interruption produces the same outsized sample as a long run of retries; Section 7.6 reports the diagnostics and their inability to identify the cause of each long sample.

### 7.5. Operational Deployment Considerations

The endpoint capability model is kept narrow. The Move contract checks ownership and a matching EndpointCap before accepting a commitment, which prevents unrelated accounts from writing commitments under a registered endpoint object, but it is not a full device identity-management system. In future versions endpoint registration could be connected to a fleet-management registry, a manufacturer certificate, or a hardware attestation root; none of that is attempted here.

The off-chain blob pointer is likewise an operational choice. In a research prototype the blob identifier can refer to decentralized storage, local experiment archives, or any content-addressed bundle. A production deployment should define retention rules, redundancy, access-control policy, and deletion behavior. The chain anchor proves that a particular record existed at commitment time only if the corresponding bundle remains retrievable or can be produced by an accountable party; the ledger cannot supply that guarantee and this design does not claim that it does.

The prototype implements key attachment, retirement, compromise reporting, and historical key resolution. Production deployment additionally requires protected key provisioning, reliable reporting, and an operational investigation policy for the possible interval between actual compromise and its report. Endpoint deactivation prevents new commitments while retaining historical evidence; it does not establish that every earlier signature was made before compromise.

The off-chain store also requires policy. A content-addressed or decentralized storage system can help ensure that the retrieved bytes match the expected bundle, but it does not decide who must retain records, for how long, or under what access-control rules. Manufacturing records may contain sensitive design or production data. A practical deployment may encrypt bundles for authorized auditors while still anchoring the hash of the ciphertext or a canonical plaintext commitment.

Connectivity is another constraint. Some endpoints may not be able to submit a chain transaction at the exact moment of actuation. The architecture can tolerate delayed anchoring if the endpoint signs records immediately and stores them locally, but delayed anchoring weakens the backfill argument. A final system should report both endpoint-side decision time and chain-side commitment time, and the policy should define the acceptable delay.

The framework can also be extended with batching. Multiple decision records can be assembled into a Merkle tree, and the root can be committed on-chain. This reduces transaction frequency while preserving record-level auditability. The tradeoff is that the auditor must obtain inclusion proofs in addition to the signed record bundle. Batching is attractive for high-rate telemetry summaries but unnecessary for low-frequency authorization decisions. Table 14 summarizes these deployment issues, their recommended handling, and their status in this paper.

### 7.6. Threats to Validity and Study Limitations

The empirical result is a microbenchmark on a single Raspberry Pi 5 and a single development workstation, not a deployment study. The benchmark uses a fixed 638-byte record, so it is suitable for checking implementation feasibility and relative scheme behavior, but not for statistical claims about a device fleet. The Raspberry Pi 5 session was verified free of thermal and under-voltage throttling and each configuration was run three times at 200 iterations with raw output archived, but a single device of a single model cannot speak to silicon-to-silicon variation.

Three measurement limitations matter. First, elapsed latency combines signing work with timer, scheduling, cache, and other host effects. The Pi sessions used a performance governor and CPU pinning, while both workstation key-effect sessions were collected without quiescing. Drift and greater-than-ten-median diagnostics identify patterns to inspect but cannot identify every interrupted signature or rule out structured noise. The earlier arm64-20260810 session remains archived as the motivation for these diagnostics and is not used for current performance estimates. Second, the two platforms used different Rust compilers (1.93.0 on the workstation and 1.97.1 on the Pi), confounding hardware with toolchain in cross-platform ratios. Third, a Raspberry Pi 5 is a Linux-class edge gateway, not a microcontroller; a Cortex-M deployment requires separate measurement. The shared message seeds, unretained keys, single observation per cell, and unresolved desktop signals further limit the variance interpretation in Section 5.2.

The implementation also measures cryptographic and audit primitives more directly than it measures a complete factory workflow. Network delay, off-chain storage latency, transaction submission time, and indexing behavior are separated from the signature benchmark. This separation makes the cryptographic comparison clearer, but end-to-end latency must be measured before claiming production performance.

The Move contract evidence is source-level, unit-test, and localnet transaction-effects evidence. The current 38 Move tests and 30 Rust tests cover registry governance, ownership, capability matching, active status, anchor length, scheme membership and revocation, migration and hybrid operation, and policy enforcement, but they are not formal verification and do not replace an adversarial Move audit. The archived Sui effects measure localnet behavior under Sui CLI 1.72.1, so they improve the evidence package but should not be generalized to public-network or production gas costs without re-measurement.

## 8. Conclusions

This paper presents Edge Attestation Lab, a crypto-agile framework for tamper-evident provenance of IoT edge actuation decisions. The framework separates complete endpoint-signed evidence from the recurring blockchain representation: decision records and signatures remain off-chain and public keys are registered per endpoint and scheme, while the chain stores a fixed 32-byte SHA-256 commitment together with bounded metadata and a one-byte signature-scheme identifier. This separation supports migration among classical and post-quantum endpoint signatures without introducing scheme-dependent signature lengths into the commitment object.

On a Raspberry Pi 5, ML-DSA-44 averaged approximately 0.65 ms across three run medians, with signing CV approximately 78% and substantial upper tails. Additional crossed measurements found no nominally significant ML-DSA key factor on the Pi, but two ML-DSA-44 excursions on the workstation. One remains significant after correction across all 72 comparisons (p≈0.0216 using archived permutation values). The cause and persistence of that association remain unresolved. The study therefore does not establish key independence, a numerical variance upper bound, or the performance of key selection on unseen messages. Tail-aware scheduling is supported by the observed latency distribution; an empirical percentile is not a worst-case timing guarantee.

On the evaluated Sui localnet, changing only the scheme identifier for the same multi-key endpoint produced identical measured commitment gas components despite a 267-fold range in off-chain signature size. Commitment gas also remained unchanged across a 3301-fold range in record size because only the 32-byte digest reached the chain. Separate endpoint configurations showed up to 5.66% variation because registered public-key length affects the cost of rewriting endpoint state on this ledger. The resulting claim is therefore precise: the architecture decouples recurring commitment cost from per-decision signature length and record size, but not from every ledger-specific property of endpoint state.

For the WSN/IoT security setting targeted by this work, the main contribution is a secure-data-management pattern for sensing-to-actuation workflows in which endpoint evidence remains independently verifiable while the ledger representation remains compact and crypto-agile. The framework makes endpoint statements tamper-evident and attributable; it does not establish physical truthfulness of firmware-reported telemetry and does not make the underlying blockchain transaction layer post-quantum secure. Future work should evaluate additional edge and microcontroller platforms, larger distinct-bundle audit workloads, delayed/offline anchoring, batching strategies, and public-network costs where deployment requires them.

## Figures and Tables

**Table 1:** Positioning against the closest prior work and two alternative approaches to post-quantum signatures on a ledger.

Dimension	This Work	Prior Work [11]	On-Chain PQC Verification [13,14]	Off-Chain Anchoring, Single Scheme [16]
Authorization mechanism	Ownership and capability checks gate who may write a commitment; execution authorization remains external	On-chain authorization predicate gates release of protected design material	Account-level transaction authentication	Sensor identity and key management, orthogonal to anchoring
Provenance granularity	Per decision: endpoint-signed record plus fixed-width anchor	Per completed task and physical object	Per transaction	Per sensor reading or batch
Endpoint signature scheme	Ed25519, ML-DSA-44/65/87, and SLH-DSA-SHA2-128f/128s	Chain-native signing; PQ migration discussed as future work	Multiple PQ schemes verified at the ledger layer	ML-DSA
Scheme agility	Governed one-byte scheme ids, per-scheme endpoint keys, hybrid operation, and in-place migration	Discussed as future work	Protocol-layer type identifier [14]	No scheme registry or measured cross-scheme migration
Recurring on-chain dependence on signature size	Signature bytes never enter the commitment path; cross-scheme invariance measured for a fixed endpoint	Not measured	Scheme-dependent verification/storage cost	Not testable across schemes in a single-scheme design
Edge evaluation	Raspberry Pi 5 cryptographic measurements plus Sui localnet effects	Functional manufacturing validation; no gas, latency, or storage measurements	Ledger-side evaluation	Sensor-to-ledger framework; different evaluation target

**Table 2:** Where each artifact of the data path lives and why.

Artifact	Location	Purpose
Provenance record	Off-chain	Full task, decision, design-file hash, telemetry, timestamp, optional previous anchor
Endpoint signature	Off-chain	Binds the endpoint key to the record bytes
Public key	Endpoint, KeyHistory, and bundle	Allows auditor verification; comparing the bundle key against the registered on-chain key is an auditor-side step
Anchor	On-chain	Fixed 32-byte tamper-evident commitment
Blob id	On-chain metadata	Pointer to the off-chain bundle
Record schema	On-chain metadata	Bounded label naming the record encoding version
Scheme id	On-chain metadata	One byte; resolves to a descriptor in the on-chain scheme registry

**Table 3:** Design goals, mechanisms, and explicit boundaries.

Design Goal	Mechanism	Boundary
Tamper evidence	SHA-256(record) committed on-chain	Detects record changes after commitment, not false original data
Source authentication	Endpoint signature over full record bytes	Depends on endpoint key protection
Scheme agility	Governed scheme registry; per-endpoint public keys held by scheme id	Auditor must support the scheme the registry names
Compact chain state	Store 32-byte anchor, not full signature	Off-chain availability remains necessary
Operational lifecycle	Endpoint object plus owner capability and active flag	Does not replace device fleet management

**Table 4:** The three counts used to describe auditing, and what each one counts. They describe different objects and are not alternative accounts of the same procedure.

Where It Appears	What It Describes	Count	On-Chain Query
Section 3.1 and Section 4.1, audit protocol	The complete audit procedure, from obtaining the commitment to checking the endpoint registration	Five steps	Required
Equation (2)	The acceptance condition as a logical conjunction, with the procedural steps collapsed	Four conditions	Required
Section 4.3, CLI verify	The offline checks the auditor CLI performs when no --commitment-id is supplied	Three checks	None

**Table 5:** Provenance record fields as implemented in the Rust prototype.

Field	Type	Audit Role
schema_version	u8	Binds the record bytes to a serialization version; participates in the anchor
task_id	String	Links record to requested actuation task
endpoint_id	String	Device-chosen identifier; the authoritative device binding is the endpoint_id field of the on-chain commitment
decision	String	Records accept, reject, start, pause, resume, complete, or error
payload_hash_hex	Hex-encoded 32-byte digest	Binds the decision to the protected design file
sensor_log_hash_hex	Hex-encoded 32-byte digest	Binds summarized telemetry to a larger log
timestamp_ms	u64	Endpoint-side event time for audit correlation
telemetry	Vector of telemetry samples	Captures reported process state around the decision
prev_anchor_hex	Optional hex digest	Allows local sequence linking

**Table 6:** Entry functions of the Move package and the checks they enforce.

Function	Checks	Effect
add_scheme	RegistryAdminCap; name, NIST level, and size bounds	Adds a SchemeDescriptor at the next sequential id
set_scheme_status	RegistryAdminCap; valid status value	Sets ALLOWED, DEPRECATED, or REVOKED and emits a status event
register_endpoint	Scheme in ALLOWED status; label and key bounds	Creates Endpoint, EndpointCap, and KeyHistory field
add_scheme_key	Owner and cap; scheme ALLOWED; no existing key for that id	Attaches a key and opens its history window
remove_scheme_key	Owner and cap; key exists	Detaches the key and closes its history window
attach_policy	Owner and cap; no policy attached	Hangs a CommitmentPolicy off the endpoint as a dynamic object field
detach_policy	Owner and cap; policy attached	Removes the CommitmentPolicy
set_endpoint_status	Owner and cap	Updates active flag and emits status event
create_commitment	Five-step chain: cap/owner/active; key held for scheme id; scheme registered and not revoked; policy admits; 32-byte anchor; bounded metadata	Creates ProvenanceCommitment and emits provenance event

**Table 7:** Experiment groups, metrics, and purpose.

Measurement	Metric	Purpose
PQC microbenchmark	Keygen/sign/verify latency and key/signature size	Quantifies scheme-level endpoint overhead over a fixed record
Edge hardware replication	Same metrics on a Raspberry Pi 5	Tests whether desktop feasibility transfers to edge-class devices
On-chain cost	Raw Sui effects and gas summary	Confirms the measured localnet cost of anchor-only entry functions and its invariance across signature schemes
Verification microbenchmark	Repeated verifications per second over one archived bundle	Confirms implementation cost only; distinct-bundle throughput, storage, and I/O are not measured

**Table 8:** Raspberry Pi 5 microbenchmark, three runs of 200 fresh keys each (600 keys per parameter set). Keygen, sign, and verify columns are means of the per-run medians. Three different medians appear in this paper and are not interchangeable: the sign median column is the mean of the three per-run medians (648.2 us for ML-DSA-44), the median of the pooled 600 samples is 593.3 us, and the per-run medians themselves range from 588 to 763 us, which is the range quoted in Section 7.4.

Scheme	PK Bytes	Sig Bytes	Keygen us	Sign Median us	Sign p95 us	Sign p99 us	Sign CV	Verify us
Ed25519	32	64	58.6	63.5	65.3	80.5	6.5%	182.5
ML-DSA-44	1312	2420	316.2	648.2	2015.1	2851.9	77.9%	104.1
ML-DSA-65	1952	3309	513.1	1094.8	3342.9	4735.6	77.9%	148.9
ML-DSA-87	2592	4627	799.0	1262.8	4377.2	6529.7	78.9%	217.9
SLH-DSA-SHA2-128f	32	17,088	460.8	10,925.2	10,985.1	11,676.8	2.1%	655.7
SLH-DSA-SHA2-128s	32	7856	29,463.8	228,839.4	229,317.7	229,647.7	0.1%	234.5

**Table 9:** Key-factor ICC estimates and archived permutation *p* values for all three sessions, each with six 40×160 runs. Values in each cell follow run order 01–06. Desktop A is arm64-20260911, Pi 5 is pi5-20260912, and Desktop B is arm64b-20260912. Drift is the largest absolute latency/order correlation within that session and scheme. Ed25519 is a comparison control, not a universal noise bound. Values are rounded here; full precision and variance components are archived.

Session/Scheme	ICC, Runs 01–06	Permutation p, Runs 01–06	Max Drift |r|
Desktop A: ML-DSA-44	0.0000/0.0000/0.0000/ 0.0070/0.0000/0.0000	0.8059/0.5995/0.9122/ 0.0003/0.5003/0.5412	0.022
Desktop A: ML-DSA-65	0.0000/0.0000/0.0000/ 0.0002/0.0000/0.0006	0.5687/0.8933/0.8659/ 0.4130/0.7074/0.3044	0.020
Desktop A: ML-DSA-87	0.0000/0.0000/0.0012/ 0.0000/0.0000/0.0000	0.5199/0.6622/0.1996/ 0.7096/0.5534/0.7762	0.027
Desktop A: Ed25519	0.0000/0.0007/0.0000/ 0.0009/0.0000/0.0000	0.5378/0.2141/0.8315/ 0.2201/0.9290/0.9080	0.292
Pi 5: ML-DSA-44	0.0018/0.0000/0.0000/ 0.0000/0.0000/0.0000	0.1077/0.9121/0.8610/ 0.9283/0.6982/0.6720	0.015
Pi 5: ML-DSA-65	0.0000/0.0000/0.0000/ 0.0000/0.0000/0.0005	0.4760/0.9267/0.6607/ 0.4994/0.6401/0.3550	0.024
Pi 5: ML-DSA-87	0.0000/0.0000/0.0000/ 0.0015/0.0000/0.0000	0.7650/0.6392/0.8499 /0.1401/0.7578/0.8867	0.018
Pi 5: Ed25519	0.0000/0.0000/0.0000/ 0.0003/0.0007/0.0025	0.4774/0.6633/0.6328/ 0.2048/0.1738/0.0427	0.097
Desktop B: ML-DSA-44	0.0000/0.0033/0.0008/ 0.0003/0.0000/0.0000	0.6711/0.0187/0.2606/ 0.3884/0.5771/0.8918	0.029
Desktop B: ML-DSA-65	0.0000/0.0016/0.0017/ 0.0000/0.0000/0.0000	0.9360/0.1316/0.1177/ 0.6750/0.6412/0.5688	0.019
Desktop B: ML-DSA-87	0.0000/0.0000/0.0002/ 0.0000/0.0000/0.0020	0.5698/0.6996/0.4318/ 0.9026/0.9452/0.0832	0.024
Desktop B: Ed25519	0.0011/0.0001/0.0000/ 0.0000/0.0006/0.0013	0.1707/0.3829/0.7555/ 0.4766/0.2951/0.1330	0.122

**Table 10:** Sui localnet transaction effects (costs in MIST; Sui CLI 1.72.1, 11 September 2026), from the same localnet session used for the on-chain experiments reported in Section 5.4, Section 5.5 and Section 5.6, measured against the package that includes the endpoint key-lifecycle record described in Section 4.2. add_scheme is reported as the range over the six registry-seeding calls. The two add_scheme_key rows carry real bundle keys of different sizes and illustrate key-dependent storage cost; registration and endpoint rewrites are also sensitive to the stored key set.

Function	Compute	Storage	Rebate	Total Gas	Created	Events
publish	1,000,000	67,662,800	0	68,662,800	4	0
add_scheme (x6, range)	1,000,000	3,921,600–4,879,200	2,753,784–4,604,688	1,209,760–2,186,208	0	1
register_endpoint	1,000,000	9,378,400	978,120	9,400,280	4	1
add_scheme_key (ML-DSA-44, 1312 B key)	1,000,000	26,653,200	6,470,640	21,182,560	0	1
add_scheme_key (SLH-DSA-SHA2-128f, 32 B key)	1,000,000	27,299,200	26,386,668	1,912,532	0	1
remove_scheme_key (SLH-DSA-SHA2-128f)	1,000,000	27,040,800	27,026,208	1,014,592	0	1
set_endpoint_status (deactivate)	1,000,000	14,804,800	14,656,752	1,148,048	0	1
set_endpoint_status (reactivate)	1,000,000	14,804,800	14,656,752	1,148,048	0	1
create_commitment	1,000,000	17,198,800	14,656,752	3,542,048	1	1

**Table 11:** Isolating the per-byte storage term on the evaluated Sui localnet. store_anchor and store_payload are otherwise identical entry functions, so the difference between them removes the payload-independent object overhead. The effective byte difference accounts for the ULEB128 length prefix a vector carries, one byte below 128 and two bytes from 128 to 16,383, so the quantity differenced is the object’s serialized length rather than its payload length. The residual is exactly zero at all eight points across a 500-fold range. Writing the measured storage charge as 7600[N+L(N)+331] MIST, where L(N) is the ULEB128 prefix length, gives 331 bytes of payload-independent charged overhead. This is an intercept for transaction storage accounting, including other charged objects, not the size of the new payload object alone or an intercept for total transaction cost.

Entry Function	Payload	Storage Cost	Difference	Effective Bytes	Per Byte
store_anchor	32 B	2,766,400	—	—	—
store_payload	64 B	3,009,600	243,200	32	7600.00
store_payload	512 B	6,422,000	3,655,600	481	7600.00
store_payload	1024 B	10,313,200	7,546,800	993	7600.00
store_payload	2420 B	20,922,800	18,156,400	2389	7600.00
store_payload	3309 B	27,679,200	24,912,800	3278	7600.00
store_payload	4627 B	37,696,000	34,929,600	4596	7600.00
store_payload	8192 B	64,790,000	62,023,600	8161	7600.00
store_payload	16,000 B	124,130,800	121,364,400	15,969	7600.00

**Table 12:** Operations and baselines used in the cost comparison, all from the same localnet session. The four baseline entry functions live in verification_baseline, a separate Move package published alongside the framework purely as measurement scaffolding, so that the edge_attestation package whose cost is reported stays byte-identical. The Ed25519 rows measure the native primitive in this configuration. They neither measure post-quantum verification nor establish a lower bound for it. Costs are in MIST; the bytes column denotes signature/payload input length, not bytes retained by every operation.

Operation	What It Isolates	On-Chain Bytes	Total Gas	vs. Anchor
store_anchor	The framework’s object shape, storage only	32	2,788,280	1.00×
store_payload	Same object, N payload bytes, no verification	2420	20,944,680	7.51×
store_payload	Same, one parameter set larger	4627	37,717,880	13.53×
store_payload	Same, just under the argument limit	16,000	124,152,680	44.53×
store_payload	An SLH-DSA-SHA2-128f signature	17,088	rejected	n/a
verify_ed25519	Native on-chain verification, storing nothing	64	1,009,880	0.36×
verify_and_ store_ed25519	Native verification plus retention	64	3,031,480	1.09×

**Table 13:** Attacks, detection mechanisms, and residual risk.

Attack	Detection or Mitigation	Residual Risk
Modify off-chain record	Anchor mismatch	Hash collision is out of scope under SHA-256 assumptions
Backfill record after failure	Ledger timestamp and event order	Requires auditors to check chain history
Impersonate endpoint	Signature plus comparison to the historically registered key	Private-key compromise remains critical
Commit under a revoked or unregistered scheme	Registry checks inside create_commitment; admin-gated scheme lifecycle (deprecation blocks new registrations only)	Registry admin capability must be protected
Repudiate an earlier record by rotating or removing the signing key	Per-key validity window in ledger time; the auditor resolves the key held when the commitment was anchored	Historical key association is preserved; attribution still assumes key protection before signing
Present a bundle signed with a key later reported compromised	The old key does not resolve at or after its report time; a replacement has its own window	Report time does not identify actual compromise or signature creation time
False firmware telemetry	Not solved by this layer	Needs hardware-rooted trust or independent sensing

**Table 14:** Deployment issues, recommended handling, and current status.

Deployment Issue	Recommended Handling	Paper Status
Key compromise	Recorded key windows, deactivation, and reporting policy	Lifecycle implemented; actual compromise time remains external
Sensitive records	Encrypt bundles; anchor stable commitments	Future extension
Offline operation	Sign immediately; anchor later with delay metadata	Future experiment
High event rate	Merkle batching with inclusion proofs	Optional extension
Fleet registry	Connect endpoint objects to manufacturer inventory	Future integration

## Data Availability

The public artifact repository is https://github.com/do0x0ob/edge-attestation-lab-artifact (accessed on 29 September 2026). The revision source code, raw measurements, protocols, and claim-to-evidence mapping are archived as the immutable release sensors-revision-2026 at https://doi.org/10.5281/zenodo.22791162 (accessed on 29 September 2026), the version DOI cited by this manuscript; https://doi.org/10.5281/zenodo.22791161 (accessed on 29 September 2026) resolves to all versions of the archive.

## Appendix A. Illustrative Manufacturing Audit Walkthrough

This section is an illustrative walkthrough of the audit procedure rather than a measured deployment study. The endpoint class and the surrounding manufacturing workflow it describes were realized in prior work [11] and exercised end to end down to a physically printed part; what follows traces how the provenance layer of this paper would be used during a dispute over such a workflow, and it makes no new experimental claim.

The motivating walkthrough is a small additive-manufacturing endpoint that receives a protected task, accepts or rejects the task, and records process telemetry while executing it. The endpoint is not assumed to be a high-performance server. It is closer to a Raspberry Pi class controller attached to a printer or another manufacturing device. The workflow therefore needs to tolerate limited compute, intermittent connectivity, and the practical need to keep on-chain writes sparse.

A typical audit begins with a disputed manufactured object. The auditor receives the object identifier, the claimed task id, and the approximate production time. The auditor queries the chain for provenance commitments created by the endpoint during that time window. For each candidate commitment, the auditor retrieves the off-chain bundle, recomputes the record anchor, verifies the endpoint signature, and checks whether the design-file hash matches the expected design file. If the endpoint used a local hash chain, the auditor also checks whether the previous-anchor sequence is continuous.

This case study exposes why decision-level records are preferable to raw high-frequency telemetry anchoring. A printer can emit many telemetry samples per second, but the accountability question usually concerns a smaller set of decision points: task accepted, print started, material loaded, layer transition, anomaly observed, job paused, job resumed, and job completed. The off-chain record can include detailed telemetry around each decision without forcing every telemetry sample into the ledger.

**Table A1:** Audit questions mapped to bundle evidence and on-chain evidence.

Audit Question	Evidence in Bundle	On-Chain Evidence
Which design file was reported?	payload_hash and task_id	Anchor binds the design-file hash to the commitment
Which endpoint recorded it?	public key, signature, endpoint_id	Endpoint registration and scheme metadata
Was the record altered?	Record bytes recomputed by auditor	32-byte anchor comparison
Was the record anchored by the required time?	Record bytes matching the anchor	Ledger timestamp and event order
Was telemetry abnormal?	Telemetry samples and sensor_log_hash	Anchor proves the telemetry statement was committed

The same procedure can be applied to other physical endpoints. In a laboratory automation device, the task could be a dispense instruction. In an agricultural controller, the task could be an irrigation or chemical-release decision. In a robotics system, the task could be a motion plan or safety stop. The common requirement is that the endpoint records a bounded decision statement, signs it, and anchors a digest that cannot be rewritten silently later.

## Appendix B. Artifact Reproducibility Details

The research artifact is organized so that the code path used for the paper can be separated from the seed application history. The standalone Move package is the primary contract package. It builds independently with sui move build under Sui CLI 1.72.1, and it contains no dependency on the older manufacturing workflow. The Rust endpoint code contains the provenance module, the auditor CLI, and the benchmark CLI. The repository README documents the build commands and distinguishes the preliminary desktop run from the Raspberry Pi 5 edge-hardware evidence. The artifact is maintained at https://github.com/do0x0ob/edge-attestation-lab-artifact (accessed on 29 September 2026); the revision archive is the immutable release sensors-revision-2026, archived at https://doi.org/10.5281/zenodo.22791162 (accessed on 29 September 2026). The manuscript uses the August microbenchmark and the three September key-effect sessions separately.

Reproducibility requires preserving raw benchmark output, not only summary tables. Table A3 lists the minimum archival item for each artifact class.

The current implementation already supports one important reproducibility property: the record bytes are fixed for the benchmark. Every scheme signs the same 638-byte serialized provenance record. This makes signature latency and verification latency comparable across schemes. The auditor CLI also supports stored JSON bundles for Ed25519, ML-DSA, and SLH-DSA, so reviewers can reproduce anchor calculation, scheme-binding checks, and signature verification from archived artifacts. Representative CLI transcripts for Ed25519, ML-DSA-44, and SLH-DSA-SHA2-128f are archived with the evidence package, along with a release-profile ML-DSA-44 batch-verification transcript. A separate transcript covers the key-lifecycle path on a live localnet in four parts: a commitment that stays verifiable across a key rotation, the same bundle rejected by an auditor that reads only the endpoint’s current key set, attribution before and refusal after a compromise report, and the fallback path for an endpoint registered before the lifecycle record existed.

The evidence package also includes a claim-to-evidence manifest. This manifest maps each central manuscript claim to source files, tests, transcripts, benchmark artifacts, or remaining raw-data requirements. Its purpose is to keep the paper’s wording aligned with what has actually been implemented and measured.

For the Move component, reproducibility includes the package hash, the Sui CLI version, the localnet identifier, the raw transaction effects, and the extracted gas summary. The archived localnet run records Sui CLI 1.72.1-94ad8ccd0ed6, the package ids for both the framework and the measurement-baseline package, the scheme-registry id, the registry admin capability id, the endpoint ids, the commitment ids, and raw effects JSON for every transaction. It also records an SHA-256 digest of the exact Move source the session was built from, which is what pins the measurement rather than the repository commit: a session may be run from a working tree, and the digest states unambiguously which bytes produced the reported gas. Although the anchor is fixed-size, transaction gas still depends on execution environment, object operations, event emission, and network version.

**Table A2:** Cryptographic and toolchain environment. Absolute latencies are properties of these implementations at their default features, not of the algorithms.

Component	Version	Note
ed25519-dalek	2.2	Default features; no target-cpu tuning
ml-dsa	0.1.1	RustCrypto; getrandom feature; deterministic signing path
slh-dsa	0.2.0-rc.5	Release candidate at the time of measurement
sha2	0.10	Anchor computation
serde_json	1.0	Record serialization; anchor stability is tied to field order
rustc (development machine)	1.93.0	macOS aarch64, release profile
rustc (Raspberry Pi 5)	1.97.1	Debian 13 aarch64, release profile
Sui CLI	1.72.1-94ad8ccd0ed6	Localnet, Move package build and transaction effects

**Table A3:** Minimum archival items per artifact class.

Artifact	Minimum Archival Item	Reason
Rust benchmark	Raw CSV plus command line	Allows plots and statistics to be regenerated
Hardware run	CPU model, OS, governor, Rust version	Explains latency differences across devices
Move package	Commit hash, CLI version, transaction effects	Makes gas and object claims auditable
Off-chain bundle	Sample record, signature, public key, scheme id, auditor transcript	Lets reviewers reproduce anchor and verification
Paper figures	Scripted plot source	Avoids manual table-to-figure drift

## References

[1] Sicari S., Rizzardi A., Grieco L.A., Coen-Porisini A. (2015). Security, privacy and trust in Internet of Things: The road ahead. Comput. Netw..

[2] Roman R., Najera P., Lopez J. (2011). Securing the Internet of Things. Computer.

[3] Nakamoto S. (2008). Bitcoin: A Peer-to-Peer Electronic Cash System. https://bitcoin.org/bitcoin.pdf.

[4] Christidis K., Devetsikiotis M. (2016). Blockchains and Smart Contracts for the Internet of Things. IEEE Access.

[5] Dorri A., Kanhere S.S., Jurdak R. (2016). Blockchain in Internet of Things: Challenges and Solutions. arXiv.

[6] Reyna A., Martin C., Chen J., Soler E., Diaz M. (2018). On Blockchain and Its Integration with IoT. Challenges and Opportunities. Future Gener. Comput. Syst..

[7] Ferrag M.A., Derdour M., Mukherjee M., Derhab A., Maglaras L., Janicke H. (2019). Blockchain Technologies for the Internet of Things: Research Issues and Challenges. IEEE Internet Things J..

[8] National Institute of Standards and Technology (2024). Module-Lattice-Based Digital Signature Standard. Federal Information Processing Standard (FIPS) 204.

[9] National Institute of Standards and Technology (2024). Stateless Hash-Based Digital Signature Standard. Federal Information Processing Standard (FIPS) 205.

[10] Josefsson S., Liusvaara I. (2017). Edwards-Curve Digital Signature Algorithm (EdDSA). RFC 8032.

[11] Hsieh Y.-Y. (2026). Implementation of a Smart-contract-based Manufacturing Authorization Framework for Parametric Design. Master’s Thesis.

[12] Xu X., Weber I., Staples M. (2019). Architecture for Blockchain Applications.

[13] Kysil R., Seres I.A., Kutas P., Kelecsenyi N. poqeth: Efficient, Post-Quantum Signature Verification on Ethereum. Proceedings of the ACM Asia Conference on Computer and Communications Security (AsiaCCS).

[14] Santos M.B., Ferrin D., Kahat R., Lodder M. (2026). Post-Quantum Blockchains with Agility in Mind. Migration and Agility in Cryptographic Systems (MAgiCS 2026); Communications in Computer and Information Science.

[15] Finlow-Bates K., Jakobsson M., Siadati H. (2026). The Cost of Quantum Resistance: A Hash-Based Commit-Reveal Alternative for Minimizing Blockchain Infrastructure Overhead. arXiv.

[16] Sezer B.B. (2026). A Post-Quantum Sensor-to-Blockchain Transaction Framework with CRQC-Aware Exposure Minimization for Next-Generation Sensor Networks. Sensors.

[17] Ducas L., Kiltz E., Lepoint T., Lyubashevsky V., Schwabe P., Seiler G., Stehle D. (2018). CRYSTALS-Dilithium: A Lattice-Based Digital Signature Scheme. IACR Trans. Cryptogr. Hardw. Embed. Syst..

[18] Bernstein D.J., Hopwood D., Hulsing A., Lange T., Niederhagen R., Papachristodoulou L., Schneider M., Schwabe P., Wilcox-O’Hearn Z. (2015). SPHINCS: Practical Stateless Hash-Based Signatures. Advances in Cryptology-EUROCRYPT 2015.

[19] Fernandez-Carames T.M. (2020). From Pre-Quantum to Post-Quantum IoT Security: A Survey on Quantum-Resistant Cryptosystems for the Internet of Things. IEEE Internet Things J..

[20] Ducas L., Kiltz E., Lepoint T., Lyubashevsky V., Schwabe P., Seiler G., Stehle D. CRYSTALS-Dilithium: Algorithm Specifications and Supporting Documentation (Version 3.1); NIST Post-Quantum Cryptography Standardization, Round 3 Submission, 2021. https://pq-crystals.org/dilithium/data/dilithium-specification-round3-20210208.pdf.

[21] Internet Engineering Task Force Adapting Constrained Devices for Post-Quantum Cryptography. Internet-Draft Draft-ietf-pquip-pqc-hsm-Constrained-06, Work in Progress, 2026. https://datatracker.ietf.org/doc/html/draft-ietf-pquip-pqc-hsm-constrained-06.

[22] Chhetri R., Dhakal S., Gautam A. (2026). Benchmarking NIST-Standardised ML-KEM and ML-DSA on ARM Cortex-M0+: Latency, Rejection-Sampling Variance, and Memory on the RP2040. arXiv.

[23] Lee J., Bagheri B., Kao H.-A. (2015). A Cyber-Physical Systems Architecture for Industry 4.0-Based Manufacturing Systems. Manuf. Lett..

[24] Sui Foundation Object Model. Sui Documentation. https://docs.sui.io/develop/sui-architecture/object-model.

[25] Mysten Labs Walrus Docs. https://docs.wal.app/.

